# Potential patterns of fall armyworm seasonal migration in West Africa modeled with atmospheric trajectory analyses

**DOI:** 10.1002/ps.70732

**Published:** 2026-03-22

**Authors:** Fan‐Qi Gao, Xue‐Yan Zhang, Rosina Kyerematen, Gao Hu, Regan Early, Jason W. Chapman

**Affiliations:** ^1^ Department of Entomology Nanjing Agricultural University Nanjing China; ^2^ Centre for Ecology and Conservation University of Exeter Penryn United Kingdom; ^3^ Department of Animal Biology and Conservation Science University of Ghana Accra Ghana

**Keywords:** fall armyworm, North Africa, Sahara Desert, seasonal migration pattern, trajectory simulation, West Africa

## Abstract

**BACKGROUND:**

The migratory invasive species fall armyworm (*Spodoptera frugiperda*, FAW) has established year‐round populations in several West African countries following its initial invasion of Africa in early 2016. However, its seasonal migratory dynamics within West Africa remain poorly understood. If FAW populations in West Africa were able to successfully cross the Sahara Desert and serve as a major source population in North Africa, this could increase the risk of further invasion into southern Europe. In this study, we used atmospheric data to perform trajectory simulations, predicting the seasonal migratory pathways of short‐distance migratory FAW individuals within West African breeding habitats and assessing the monthly probabilities of long‐distance migrants departing from West Africa successfully crossing the Sahara Desert.

**RESULTS:**

The results indicate that from May to September, the vast majority of short‐distance migrants (>70%) remained within West African breeding habitats, whereas in other months, a larger proportion of individuals were blown into the Atlantic Ocean. Moreover, short‐distance migrants exhibited clear seasonal movement patterns within West Africa: shifting southwestward from January to May, turning northeastward in June and July, and returning southwestward from August to December. Long‐distance migrants had an extremely low success rate (≤0.3%) of crossing the Sahara Desert, which occurred only between February and April each year.

**CONCLUSION:**

This study revealed the seasonal migration patterns of FAW within West Africa, providing important insights for predicting regional outbreak risks and optimizing management strategies in the region. © 2026 The Author(s). *Pest Management Science* published by John Wiley & Sons Ltd on behalf of Society of Chemical Industry.

## Graphical abstract

Migratory fall armyworms in West African breeding habitats showed clear seasonal movements: shifting southwest from January to May, turning northeast in June to July, and returning southwest from August to December.

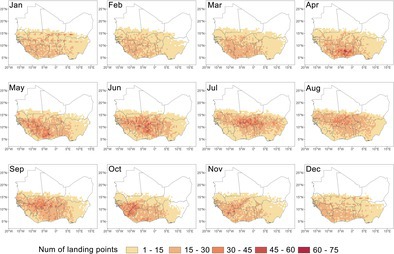

## 1 INTRODUCTION

Insect migration is a large‐scale natural phenomenon, with many species undertaking long‐distance, multi‐generational seasonal migrations.^1^, ^2^ In higher latitude regions, migratory insects typically perform directional migrations, moving between seasonal habitats to exploit temporary resources and thereby maximizing reproductive success.^3^, ^4^, ^5^ However, in tropical and subtropical low‐latitude regions, where annual temperature fluctuations are small and food resources are widely distributed year‐round, the necessity for such directional migration may be reduced.^6^ This may result in migratory behaviors that differ from those observed at higher latitudes.^6^, ^7^, ^8^ In contrast to temperate regions such as East Asia and North America where migration patterns have been intensively studied, our knowledge of insect migration in low‐latitude regions—particularly regarding the seasonal migration patterns of major agricultural pests—remains somewhat limited and lacks systematic understanding.

The fall armyworm (FAW, *Spodoptera frugiperda*) is a migratory noctuid pest native to the Americas, with a preference for host plants such as corn, rice, and sorghum.^9^, ^10^ In its native range in the USA, FAW expands from its year‐round breeding regions in southern Texas and Florida each spring and undertakes seasonal northward migrations, leading to outbreaks across most states east of the Rocky Mountains and into southern Canada.^10^, ^11^ In 2018, FAW was first detected in Asia, and within just 2 years it established a seasonal migration pattern similar to that in the USA: overwintering in the Indochinese Peninsula and South China, then migrating northward during the warmer seasons to reach Northeast China, Japan, and the Korean Peninsula.^12^, ^13^, ^14^, ^15^, ^16^, ^17^ Studies have shown that in these higher latitude regions of the USA and China, FAW populations consistently show strong migratory capacity and directed migrations that track seasonal resources.^8^, ^10^, ^18^

FAW was first reported in West Africa, specifically Nigeria and Ghana, in January 2016, and rapidly spread across sub‐Saharan Africa within the next 2 years.^19^, ^20^, ^21^ The annual economic losses caused by FAW in major corn‐producing countries in Africa are estimated at between $2.2 billion and $5.5 billion, with continent‐wide losses reaching up to $9.4 billion.^22^ Modeling studies have predicted that most sub‐Saharan countries provide year‐round suitable habitats for FAW,^23^ a prediction supported by recent evidence of its permanent breeding populations in several West African countries such as Ghana and Burkina Faso.^21^, ^24^ Our previous research indicates that, despite the low latitude and the widespread distribution of suitable year‐round habitats for FAW development in West Africa, a FAW population from Ghana has retained a high migratory capacity, which likely facilitates its timely escape from deteriorating habitats.^7^ In West Africa, the distribution of suitable habitats is largely influenced by the alternation of rainy and dry seasons and is strongly associated with unpredictable rainfall patterns. Therefore, we propose that regular directional migration may not confer advantages in this region. Instead, FAW in West Africa is more likely to exhibit nondirectional migration, relying on randomly oriented high‐altitude winds to maximize displacement.^6^, ^7^ Although the directional seasonal migration of FAW in the Americas and Asia has been widely investigated and modeled,^11^, ^25^, ^26^ studies on the potential nondirectional migration dynamics in low‐latitude regions are still lacking.

Recently, FAW have also been detected in parts of North Africa, primarily in Northeast Africa such as Sudan and Egypt.^27^, ^28^ Their arrival in this region was likely either from source populations in the Middle East and/or Arabian Peninsula region,^29^ or source populations in East Africa (e.g., Ethiopia, Kenya), which could have expanded to Northeast Africa by moving along the Nile River corridor without having to cross the vast Sahara Desert.^21^ By contrast, FAW populations from West Africa would need to successfully traverse the desert to reach potential breeding habitats in Northwest Africa, such as the coastal regions of Morocco, Tunisia, and Algeria.^23^

There have been no confirmed records of FAW in Northwest Africa, but our previous studies have found that approximately 10% of the West African FAW population are capable of sustained overnight flight, indicating the presence of individuals with strong migratory ability and, possibly, the potential to traverse the Sahara Desert.^7^ Insect migration across the Sahara Desert may seem an exceptionally challenging prospect, but at least one specie is known to do this annually. A study combining population and trajectory modeling demonstrated that painted lady butterflies (*Vanessa cardui*) undergo regular trans‐Saharan migrations, making use of favorable winds to assist their crossing.^30^ More recently, whole‐genome resequencing of painted ladies from both sides of the Sahara has confirmed the existence of long‐distance migrants capable of crossing the desert.^31^ However, compared with the painted lady, a larger, diurnal migratory insect capable of self‐powered flight at approximately 6 m s^−1^, FAW is smaller and has a lower self‐powered flight speed of about 3 m s^−1^, meaning its successful crossing of the Sahara may rely more heavily on favorable high‐altitude winds.^30^, ^32^

This study aims to address two questions: (i) What are the seasonal migration dynamics of FAW populations within its breeding region in West Africa? Addressing this question will enhance our understanding of FAW migration behavior in low‐latitude regions and provide a scientific basis for pest forecasting and management in West Africa; and (ii) Do FAW populations in West Africa have the potential to utilize high‐altitude winds to successfully cross the Sahara Desert and reach potential habitats in Northwest Africa, and during which months is such a crossing most likely? If our results indicate that West African FAW are capable of making this trans‐Saharan journey, Northwest Africa could serve as a critical stepping stone for their further invasion into Southwest Europe.

## 2 MATERIALS AND METHODS

### 2.1 Study area and FAW takeoff location selection

**Figure 1:**
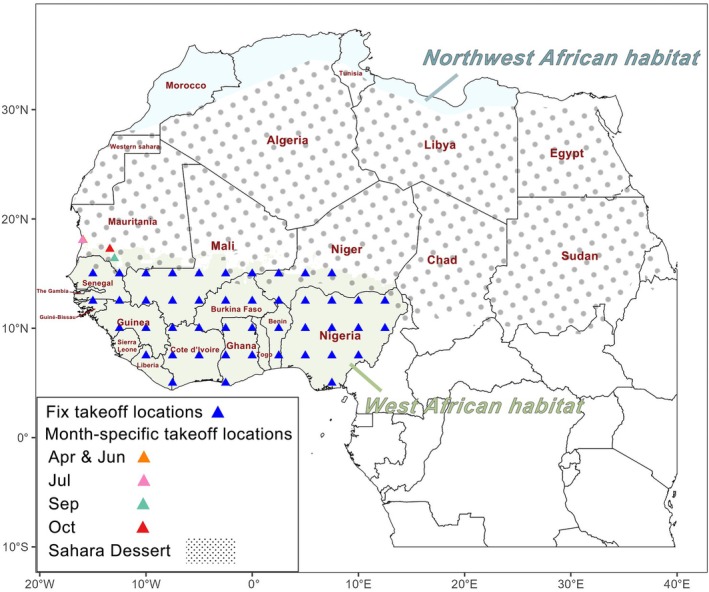
Study area and distribution of fall armyworm (FAW) takeoff locations. Green‐shaded area represents the year‐round habitat of FAW in West Africa. The gray‐dotted area indicates the Sahara Desert, and the blue‐shaded area denotes the potential habitat of FAW in Northwest Africa. Blue triangles indicate locations with daily FAW departures in the model, and triangles in other colors represent locations with departures in specific months only. The takeoff locations in April, June and July are adjacent, resulting in overlap in the figure.

The study area encompasses three regions: the year‐round breeding habitat of FAW in West Africa, the Sahara Desert, and the potential suitable breeding habitat in Northwest Africa (Fig. 1). First, based on the predictions from previous species distribution models, we extracted the area in West Africa that is suitable for FAW survival throughout the year, defined as the year‐round habitat.^23^ This area corresponds to the overlapping suitable regions from the model prediction across all months. Subsequently, we downloaded the boundary data of the Sahara Desert from the Natural Earth database (https://www.naturalearthdata.com/) and, based on its extent, delineated the non‐desert regions in northwestern Africa as potential suitable habitat for FAW.^33^

To simulate migratory trajectories, we evenly placed 45 takeoff locations within the year‐round habitat in West Africa, spaced at 2.5° intervals in both latitude and longitude (Fig. 1). From each takeoff location, particles were released daily into the atmospheric model, where they were driven by the wind fields to simulate potential FAW migration pathways. In addition, we collected five FAW distribution points from the FAMEWS platform (http://www.fao.org/fall‐armyworm/monitoring‐tools/famews‐global‐platform/en/) that are situated within West African countries but outside the suitable habitat.^34^ Each of these points was used as a takeoff location in the atmospheric model for the month in which FAW individuals were recorded (Fig. 1).

### 2.2 Simulation of migratory trajectories

The study period spanned 1 January 2021 to 30 December 2023. We first obtained the Final Analysis (FNL) data provided by the National Centers for Environmental Prediction for this period, which served as the initial and boundary atmospheric conditions for the trajectory simulations. This global data set includes multiple meteorological variables such as wind speed, temperature, and precipitable water, with a temporal resolution of 6 h and a spatial resolution of 1°. We then imported the FNL data into the Weather Research and Forecasting (WRF) model (v.3.8, www.wrf-model.org)^35^ to generate hourly atmospheric fields with higher temporal resolution for use as input to the trajectory model.^36^ Details of the WRF configuration are provided in Supporting Information, Table S1.

Based on the WRF‐derived meteorological fields, we ran forward trajectory simulations from each designated takeoff location to estimate the potential landing areas of FAW from West Africa. The simulation settings were based on six observed behavioral characteristics of FAW. (i) Takeoff time: as in other noctuid moths, FAW typically initiates flight around dusk, we therefore set the takeoff time to 19:00 local time, corresponding to sunset in West Africa. (ii) Self‐powered flight speed of 3 m s^−1^.^26^, ^32^ (iii) Flight altitude: given that noctuid moths often migrate in low‐level jet streams,^37^ six initial flight altitudes were selected to cover the likely altitude range, namely 750, 1000, 1250, 1500, 1750, and 2000 m above sea level. (iv) Temperature threshold: flight was terminated if the temperature at flight altitude fell below 13.8 °C.^38^, ^39^ (v) Flight direction: because the preferred flight orientation of African FAW populations remains unclear, we assumed that their self‐powered flight direction aligns with the prevailing wind. (vi) Flight duration: to investigate the seasonal migration dynamics within West African habitats, we simulated short‐distance flights with a duration of 5 h per night over three consecutive nights.^7^, ^18^ To evaluate the potential for long‐distance migration across the Sahara Desert, we simulated a single continuous 36‐h flight, considering that FAW individuals landing in the desert during the day may perish due to extreme heat.^32^

Finally, six simulated particles were released at 19:00 each day during the study period from each takeoff location, corresponding to the six defined altitudes. Each particle was assigned to the above flight parameters and tracked within the WRF‐derived wind field to simulate its migratory trajectory. Notably, not all takeoff locations were active every day; those located outside the year‐round West African breeding habitat were only used in specific months (Fig. 1).

### 2.3 Data analysis

All trajectory data generated from the trajectory model were processed in R. For short‐distance migration (5 h of flight per night for three consecutive nights), we extracted the monthly endpoints of all trajectories and calculated the proportion of landings both on land and within the year‐round West African breeding habitat. Using the ‘terra’ package,^40^ the trajectory endpoints that landed within the West African habitat each month were converted into raster maps with a spatial resolution of 0.5°, to visualize their spatial distribution. For clusters of landings, we performed endpoint tracing analysis to identify their corresponding takeoff locations, representing the simulated source regions.

For the long‐distance migration scenario, we calculated the number and proportion of trajectories that successfully crossed the Sahara Desert and reached the Northwest African potential habitat each month. We then mapped these successful trajectories along with their takeoff locations.

### 2.4 Seasonal wind field mapping

We used ERA5 meteorological data and R to plot the mean seasonal nocturnal (19:00–00:00) high‐altitude wind fields at 850 hPa (~1500 m above sea level) over the West African FAW breeding habitat for 2021–2023, to help interpret the migration trajectory results.^41^

## 3 RESULTS

### 3.1 Seasonal proportion of short‐distance trajectory endpoints on land and in West African breeding habitats

**Figure 2:**
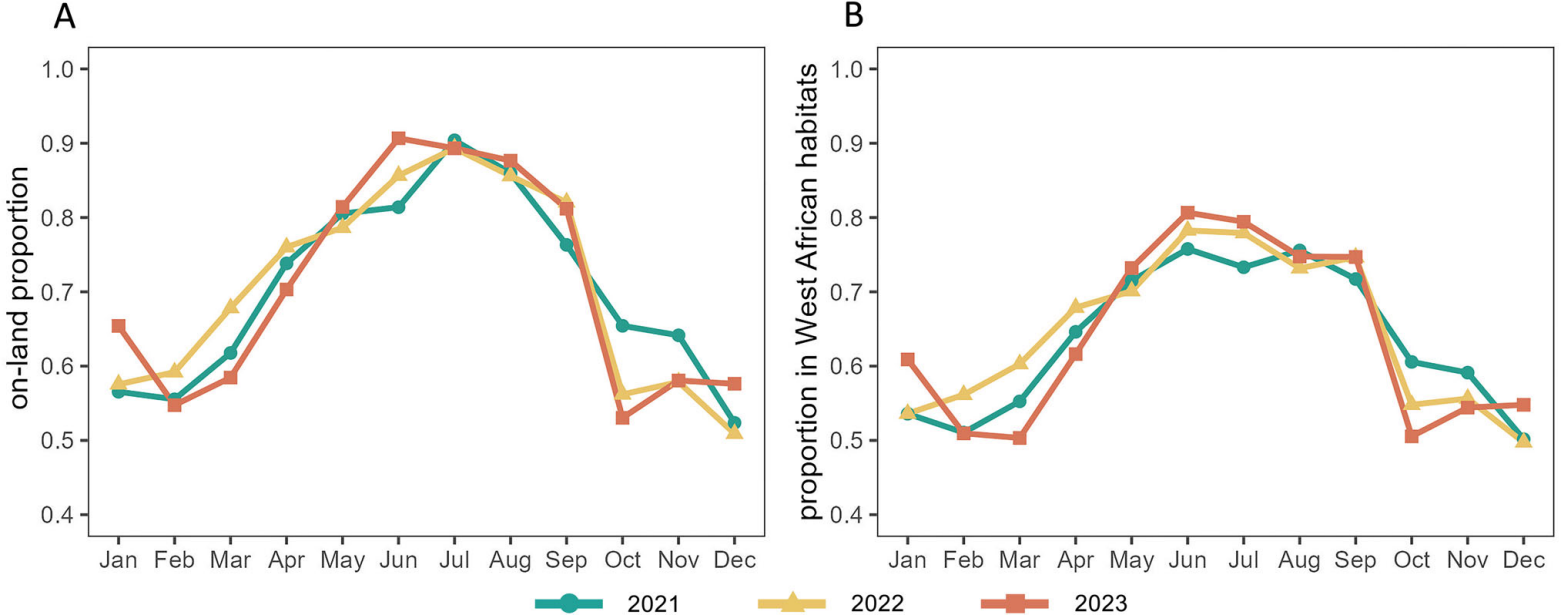
Proportion of trajectory endpoints of short‐distance migratory fall armyworm on land (A) and within West African habitats (B) across months.

Short‐distance migratory individuals were defined as FAW flying 5 h per night for three consecutive nights. During spring and summer (April–September), the proportion of trajectory endpoints falling on land was relatively high (exceeding 0.7), whereas in autumn and winter (October–March), a larger proportion ended over the ocean (Fig. 2(A); Supporting Information, Fig. S1). From May to September, the percentage of trajectory endpoints remaining within West African breeding habitats peaked, with more than 70% of endpoints located in the region (Fig. 2(B); Supporting Information, Fig. S1). Together, these results suggest that most land‐based trajectory endpoints during May to September were concentrated within FAW breeding habitats in West Africa.

### 3.2 Seasonal distribution of trajectory endpoints for short‐distance migratory FAW within West African breeding habitats

**Figure 3:**
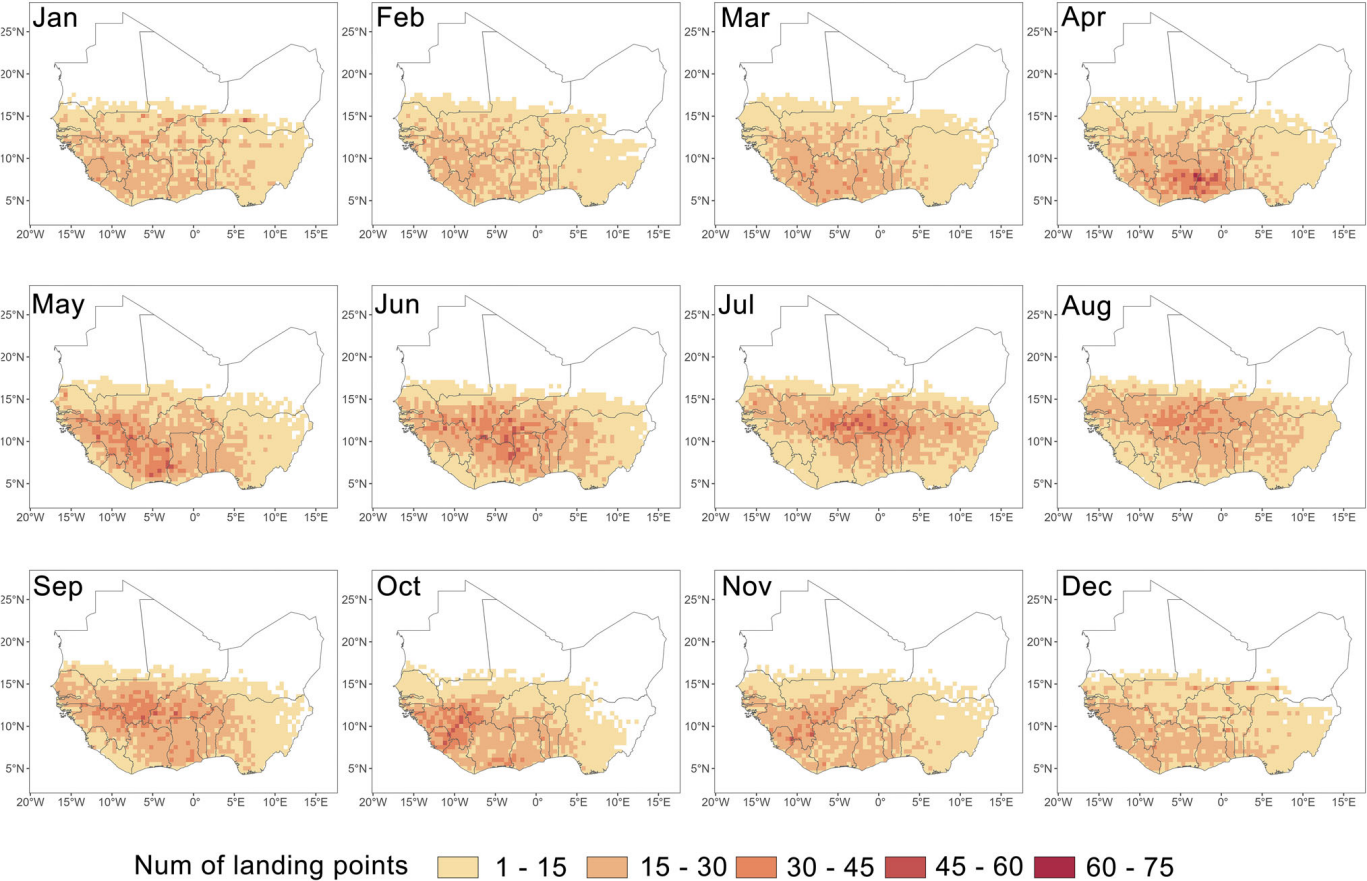
Spatial distribution maps of trajectory endpoints for short‐distance migratory fall armyworm within West African breeding habitats across months (aggregated results for 2021–2023).

**Figure 4:**
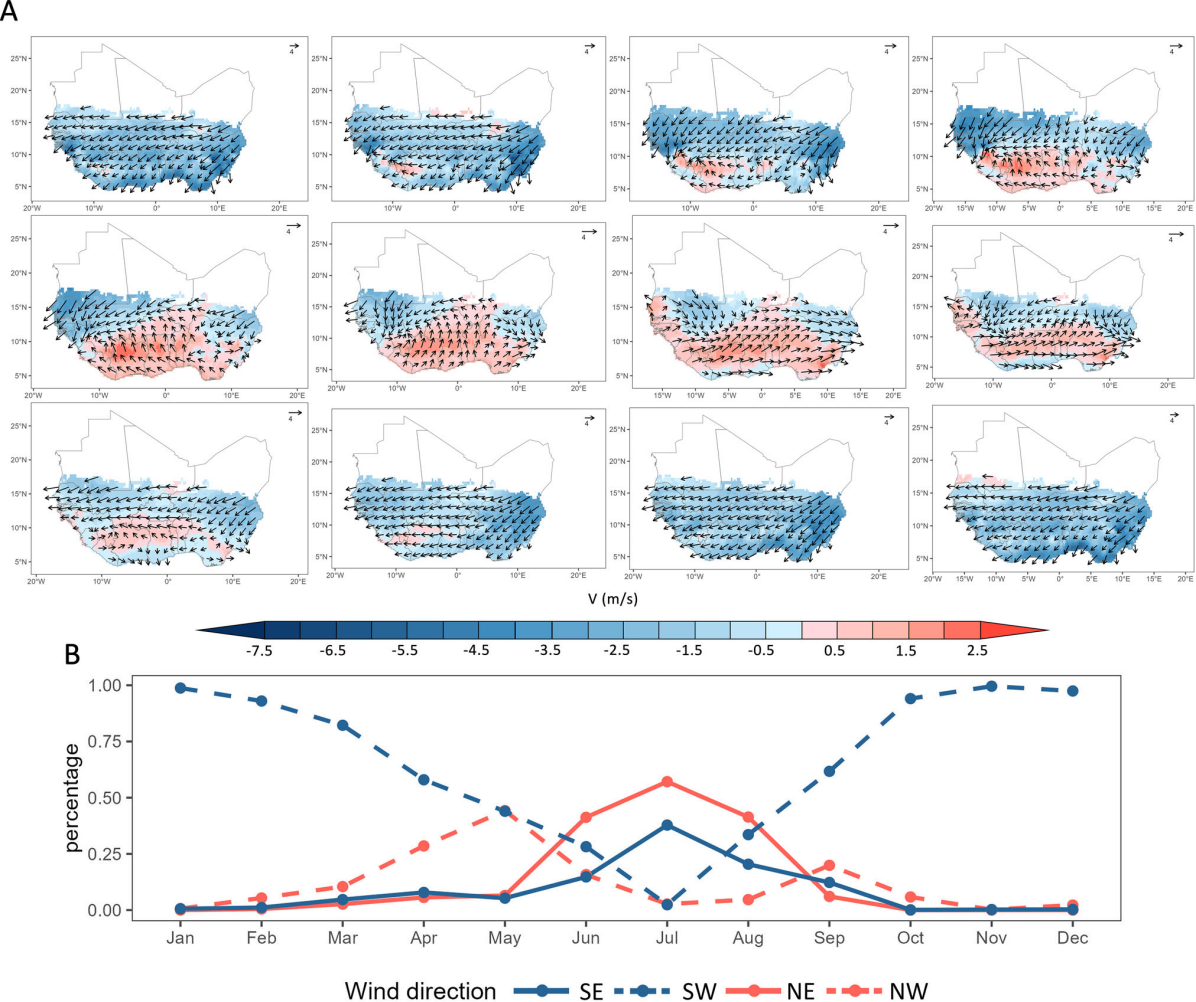
Night‐time atmospheric conditions at 850 hPa (~1500 m above sea level) over West African breeding habitats across different months. (A) Monthly wind field maps. (B) Monthly downwind direction line charts. The black arrows on the map indicate the direction and speed of the downwind vectors. The colors overlaid on the map represent the speed of the v‐component (that is, the north–south component of the wind): red (positive values) indicates winds with a northward component, while blue (negative values) indicates winds with a southward component. SE, SW, NE and NW represent winds blowing toward the southeast, southwest, northeast and northwest, respectively.

The spatial distribution of FAW trajectory endpoints within the West African breeding habitats exhibited a seasonal shift: trajectories were directed southwestward from January to May, turning northeastward in June and July, and then shifting back southwestward from August to December (Fig. 3). Notably, during June and July, the high‐altitude wind field over the FAW breeding habitat within West Africa was dominated by southwesterly winds (blowing toward the northeast), which closely corresponded to the observed directional shift in endpoints locations during the same period (Fig. 4).

### 3.3 Seasonal distribution of simulated source areas of short‐distance migratory FAW within West African breeding habitats

**Figure 5:**
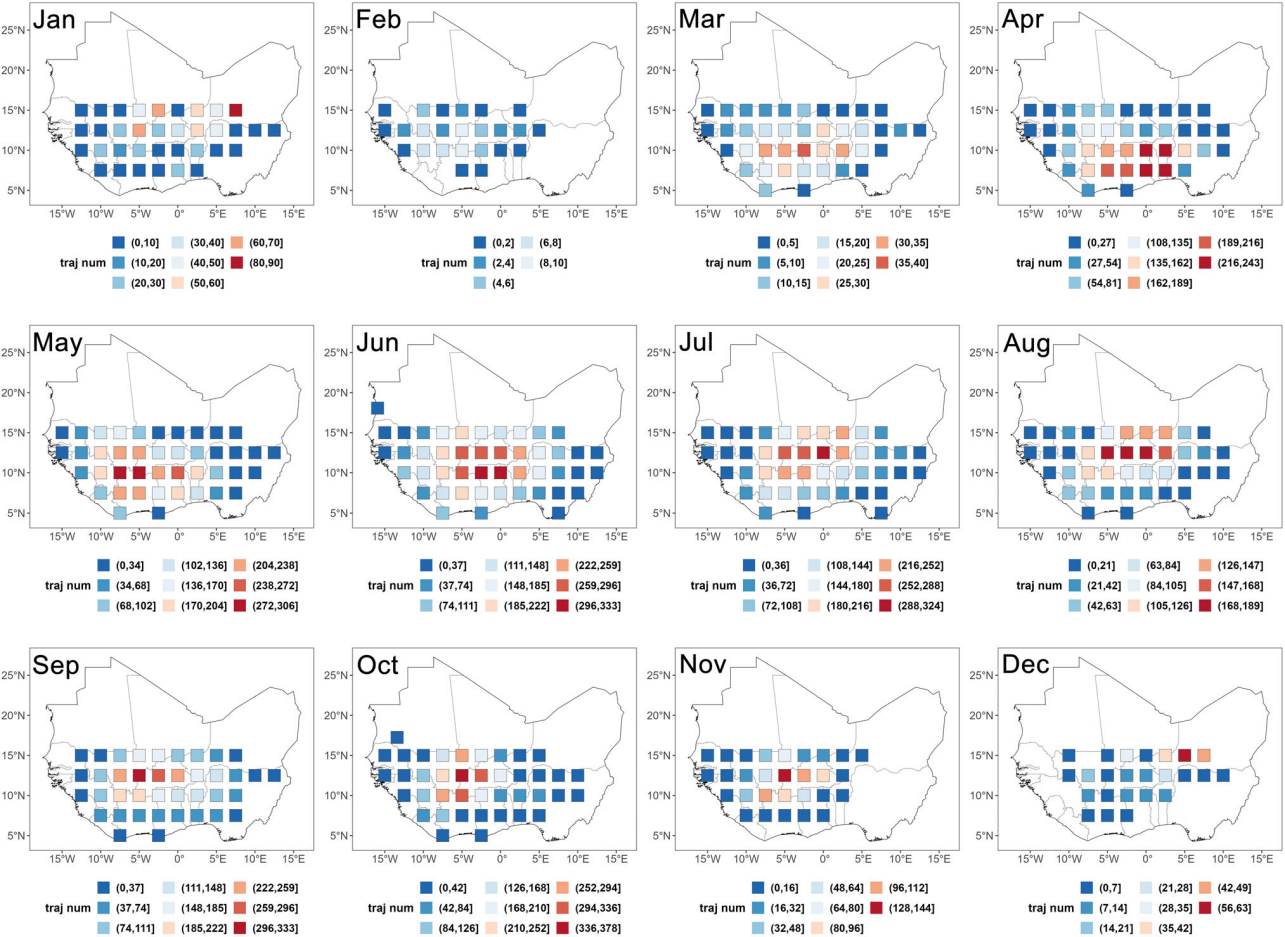
Monthly distribution of takeoff locations (simulated source areas) corresponding to potential outbreak hotspots. The different colors represent the trajectory density of takeoff locations, ranging from low (blue) to high (red). These trajectories specifically refer to those arriving at the potential outbreak hotspots.

We conducted endpoint tracing analysis for the areas in Fig. 3 with more than 30 trajectory endpoints (shown in dark orange to dark red), which represent potential outbreak hotspots, to identify their corresponding takeoff locations; that is, the simulated source areas. The spatial distribution of these source areas exhibited clear seasonal variation. From November to February, the source areas were mainly concentrated in the central and western parts of the FAW breeding habitat of West Africa, with little activity in the east (e.g., Nigeria). From March to July, the source areas gradually expanded eastward and then shifted back westward after July (Fig. 5). Throughout the year, source areas with higher trajectory densities were generally concentrated in central West Africa. Among them, Ghana, Côte d'Ivoire, and Burkina Faso emerged as key source regions during the period from May to August (Fig. 5)—when short‐distance migratory FAW individuals had greatest possibility of landing within West African breeding habitat (Fig. 2). It is important to note that although each takeoff point in the simulation was assigned the same trajectory density (one takeoff per day at each altitude), the ‘higher trajectory densities’ referred to here do not indicate that these source areas had more total trajectories. Rather, they reflect a higher density of trajectories from these source areas that successfully reached the outbreak hotspots.

### 3.4 Seasonal identification of long‐distance FAW trajectories crossing the Sahara Desert

**Figure 6:**
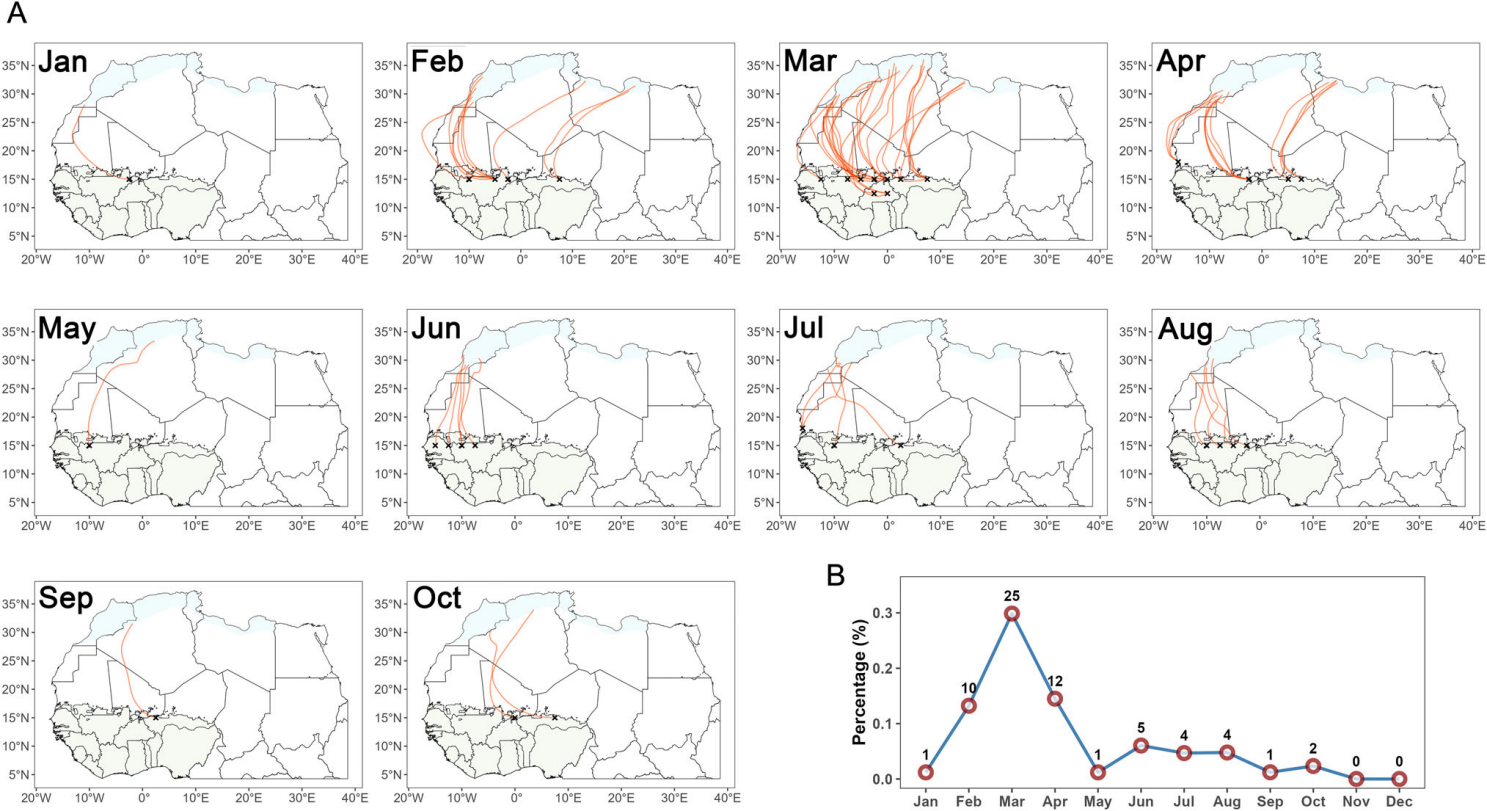
Migration trajectories of long‐distance migratory fall armyworm (FAW) crossing the Sahara Desert and reaching potential habitats in Northwest Africa in different months. (A) Monthly map of trajectories. (B) Line chart of the monthly proportion of successful trajectories. The green area represents the West African FAW breeding habitat, and the blue area indicates non‐desert regions in Northwest Africa (potential habitats).

Using a long‐distance migration scenario involving a single 36‐h continuous flight, we evaluated the monthly proportion and number of trajectories that successfully reached the potential habitats in Northwest Africa from the West African breeding areas. The results show that the success rate of crossing the Sahara Desert is extremely low. Only a few trajectories (≤0.3%) managed to arrive in non‐desert areas of Northwest Africa (Morocco, Algeria, Tunisia, and Libya) during February to April (Fig. 6(A); Supporting Information, Fig. S2). In other months, the success rate dropped below 0.1%, with no successful crossings observed in November and December (Fig. 6(B); Supporting Information, Fig. S2). All successful trajectories originated from the northern edge of the West African FAW habitat, indicating that only FAW populations near the southern margin of the Sahara have the geographic potential to complete this long‐distance migration.

## 4 DISCUSSION

In this study, we combined flight behavior parameters of the FAW with meteorological data from 2021 to 2023 to conduct two trajectory‐based analyses to: (i) examine the seasonal migration dynamics of short‐distance migratory individuals within the West African breeding habitat; and (ii) assess whether long‐distance migratory individuals originating from West Africa have the capacity to cross the Sahara Desert and reach potential habitats in Northwest Africa. The results showed that most short‐distance migrants remained within the breeding habitat in West Africa between May and September, with a relatively low probability of being blown into the Atlantic Ocean (<40%). The spatial distribution of trajectory endpoints within West African habitat exhibited a clear seasonal pattern: shifting southwestward from January to May, turning northeastward during June and July, and returning southwestward from August to December. Ghana, Côte d'Ivoire, and Burkina Faso were the major source regions associated with areas of high trajectory endpoint density. For long‐distance migratory individuals, only a very small proportion (≤0.3%) of those located near the southern edge of the Sahara Desert were likely to successfully cross the desert during the period from February to April each year.

Trajectory analysis indicated that most short‐distance migratory individuals remained within the West African breeding habitat between May and September. Meanwhile, previous studies based on species distribution models have shown that habitat suitability in this region also reaches its annual peak during this period.^23^ Therefore, we infer that the risk of FAW outbreaks is relatively high during this stage, and that the outbreak areas may exhibit a dynamic spatial pattern—first shifting northeastward and subsequently moving back southwestward in response to the seasonal variation in the direction of migration trajectories.

When exploring the seasonal migration dynamics of FAW in West Africa, we assumed a uniform distribution of source within habitats (takeoff points) in the trajectory analysis because of the lack of detailed information on local pest occurrences across the region. In reality, differences in habitat suitability among regions, combined with the spatial variation in the distribution of FAW trajectory endpoints, may lead to an aggregated distribution of source populations. However, these factors have not been incorporated into our trajectory model. Our simplified assumption may lead to deviations between the simulation results and actual conditions. Future studies could consider integrating species distribution models to predict potential habitats and selectively identifying takeoff points for the following month based on the distribution of landing sites from the previous month.^26^

Although this study demonstrates that FAW populations located at the northern edge of their West African breeding habitat have the potential to cross the Sahara Desert between February and April, the actual likelihood of such events is extremely low. Moreover, the environmental suitability of this region during that period is poor, making it unlikely to support large‐scale migration.^23^ Therefore, the possibility that FAW from West Africa could serve as a significant source population for Northwest African regions such as Morocco, Algeria, Tunisia, and Libya is limited. By contrast, FAW from East Africa can spread along the Nile River valley into Northeast African regions such as Egypt and Sudan,^21^ and nearby Middle Eastern regions such as Israel and Lebanon may also provide supplementary sources.^42^, ^43^ Although previous studies have set takeoff points in both eastern and western regions of North Africa to simulate potential FAW invasion routes into southern Europe,^44^ we suggest that the most likely pathway is via Northeast Africa, because stable source populations are currently not present in Northwest Africa.

The painted lady has been shown to successfully cross the Sahara and, in certain years, occur in large numbers in West Africa, leading to spectacular immigration events to Europe.^30^, ^31^ In addition to having a higher flight speed than FAW,^32^, ^45^ the painted lady has a much broader host range.^46^ It can utilize annual herbaceous plants in arid regions to build large populations and extend its potential range in West Africa into more northerly dry areas where FAW cannot survive, thereby reducing the distance required to cross the Sahara.^30^, ^47^

This study used trajectory simulation techniques to predict the seasonal migratory patterns of FAW within its West African habitat. From a meteorological perspective, the results offer critical insights for monitoring FAW outbreak dynamics in the region. Furthermore, the findings provide a valuable reference for understanding the spatiotemporal variability of migratory pests in tropical and subtropical regions at low latitudes.

## CONFLICT OF INTEREST

The authors declare no conflicts of interest.

## Supporting information


**Data S1.** Supporting Information.

**Data S2.** Supporting Information.

## Data Availability

Data sharing not applicable to this article as no datasets were generated or analysed during the current study.
