# Supplementary material for: Potential patterns of fall armyworm seasonal migration in West Africa modeled with atmospheric trajectory analyses
Source: Pest Manag Sci. 2026 Mar 22;82(7):6509–17. doi: 10.1002/ps.70732 (PMC13240689; doi:10.1002/ps.70732)
Supplement: Supplementary file 1 — Data S1. Supporting Information. [file PS-82-6509-s001.docx]

**Potential patterns of fall armyworm seasonal migration in West Africa modelled with atmospheric trajectory analyses**

**Fan-Qi Gao^1,2^, Xue-Yan Zhang^2^, Rosina Kyerematen^3^, Gao Hu^2^, Jason W. Chapman ^1,2^**

1 Centre for Ecology and Conservation, University of Exeter, Penryn, Cornwall TR10 9FE, United Kingdom

2 Department of Entomology, Nanjing Agricultural University, Nanjing 210095, China

3 Department of Animal Biology and Conservation Science, University of Ghana, Legon, Accra P. O. Box LG 67, Ghana

*To whom correspondence may be addressed. Email: [fg362@exeter.ac.uk](mailto:fg362@exeter.ac.uk)

**Table S1.** Parameter Settings of the WRF Model

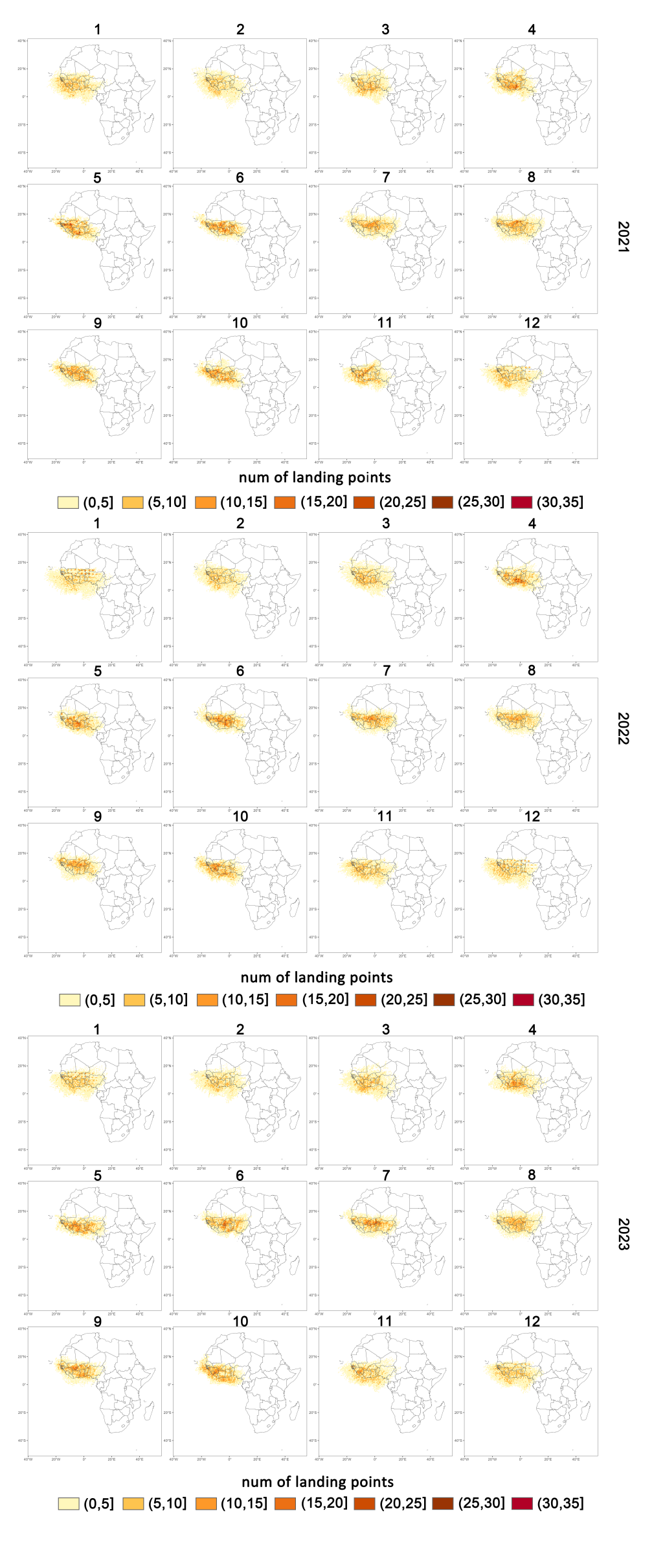


**
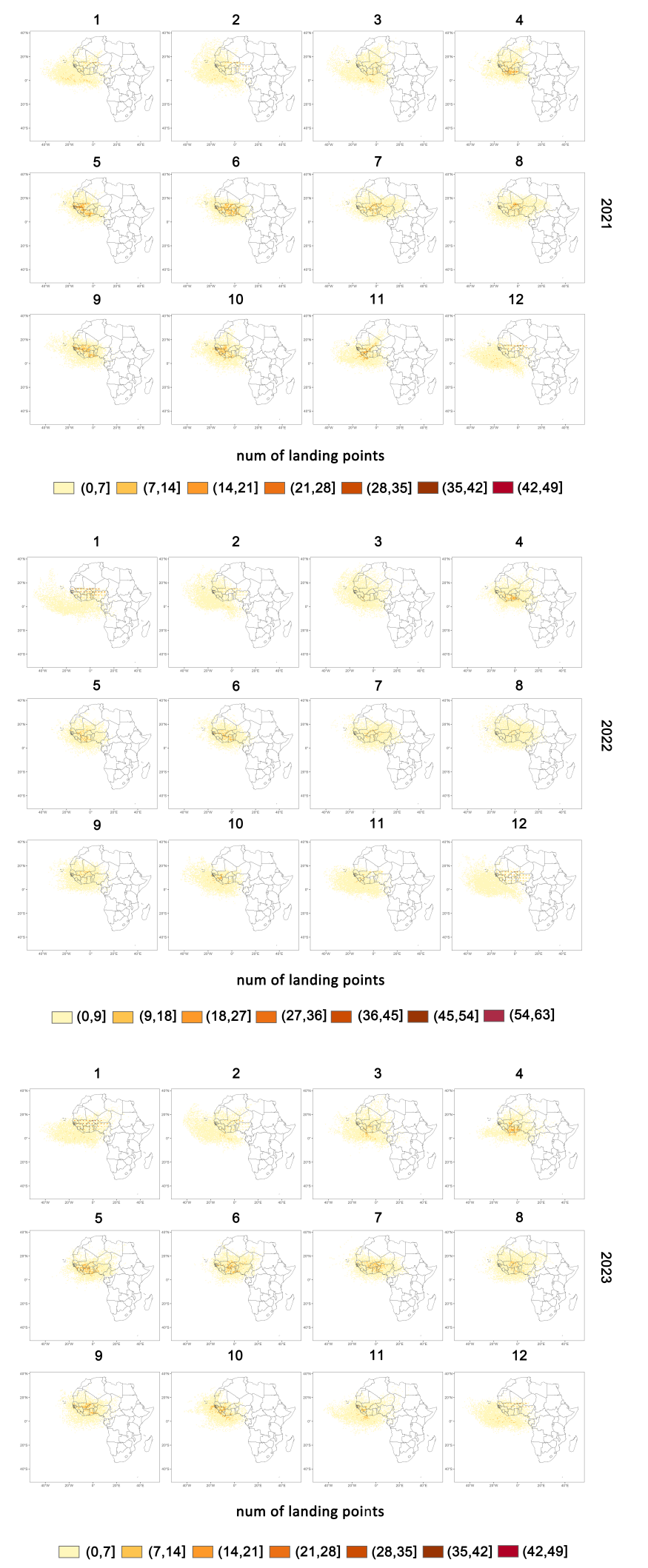
Figure S1.** Monthly distribution of landing sites for short-distance migratory FAW (5 h per night for 3 consecutive nights).

**Figure S2.** Monthly distribution of landing sites for long-distance migratory FAW (a single 36-hour continuous flight).
