# Supplementary material for: Potential patterns of fall armyworm seasonal migration in West Africa modeled with atmospheric trajectory analyses
Source: Pest Manag Sci. 2026 Mar 22;82(7):6509–17. doi: 10.1002/ps.70732 (PMC13240689; doi:10.1002/ps.70732)
Supplement: Supplementary file 2 — Data S2. Supporting Information. [file PS-82-6509-s002.zip › 3_sup 2.zip!/Supplementary Material2/raw data/ne_10m_admin_0_countries_chn.README.html]

Natural Earth » Blog Archive » Admin 0 – Countries - Free vector and raster map data at 1:10m, 1:50m, and 1:110m scales 


Free vector and raster map data at 1:10m, 1:50m, and 1:110m scales

Search for:

- Home
- Features
- Downloads
- Blog
- Issues
- Corrections
- About

---

## Admin 0 – Countries

*There are **258 countries** in the world. Greenland as separate from Denmark. Most users will want this file instead of sovereign states, though some users will want map units instead when needing to distinguish overseas regions of France.*

*Natural Earth shows **de facto** boundaries by default according to who controls the territory, versus *de jure*. Optional point-of-view (POV) variants are available for several dozen countries in the next section.*

Download countries (4.7 MB) version 5.1.1

Download without boundary lakes (4.87 MB) version 5.1.1

**About**

Countries distinguish between metropolitan (homeland) and independent and semi-independent portions of sovereign states. If you want to see the dependent overseas regions broken out (like in ISO codes, see France for example), use map units instead.

Each country is coded with a world region that roughly follows the United Nations setup.

Countries are coded with standard ISO and FIPS codes. French INSEE codes are also included.

Includes some thematic data from the United Nations (1), U.S. Central Intelligence Agency, and elsewhere.

**Disclaimer**

Natural Earth Vector draws boundaries of countries according to defacto status. We show who actually controls the situation on the ground. Please feel free to mashup our disputed area themes to match your particular political outlook.

**Known Problems**

None.

**Version History**

- 5.1.1
- 5.1.0
- 5.0.1
- 5.0.0
- 4.1.0
- 4.0.0
- 3.1.0
- 3.0.0
- 2.0.0
- 1.4.0

The master changelog is available on Github »

This entry was posted
on Friday, September 25th, 2009 at 2:13 am and is filed under 10m-cultural-vectors, Featured on Homepage.
You can follow any responses to this entry through the RSS 2.0 feed.
Both comments and pings are currently closed.


Comments are closed.

- Subscribe: Entries | Comments
- ## Search

  Search for:
- ## Links

  - NACIS
- ## Tags

  10m
  50m
  90
  180
  admin-0
  bjorn
  bounding box
  browser
  change log
  corrections
  countries
  Downloads
  error
  extent
  ext js
  forums
  geoext
  hans
  imagery
  import
  mapnik
  maptiler
  map tiles
  marine boundary
  national parks
  new data
  nsd
  openlayers
  physical labels
  pngng
  populated places
  raster
  terrestrial hypsography
  tfw
  thematic mapping
  themese
  tif
  tilecache
  tiles
  time zones
  towns
  transportation
  update
  visitors
  world file
- ## Recent Comments

  - Aligning Natural Earth Geojson and Raster to render in D3 – BBSCODE on 1:50m Shaded Relief
  - QGIS a Mapping Tool – April Dahn on 1:50m Shaded Relief
  - More Mapping with QGIS – History 502 on 1:50m Shaded Relief
  - buy ivermectin 12 mg tablets on Download URLs – double slash
  - Building A Beautiful And Clear Map From Massive, Complex Data – Slacker News on 1:10m Shaded Relief
- ## Recent Forum Topics

  - Natural Earth in Wagner VII
    by Hugo Ahlenius
  - Downloads are 404ing
    by Nathaniel
  - Disputed Territories: "type" field
    by alykat
  - ISO code confusion
    by nth
  - Bad ADM1NAME, encoding in version 3.0.0 and missing diacritics in NAME
    by pfunes
  - U.S. County Shape File
    by gzingsheim
  - Projection / Proportion / Compatibility?
    by Liquidized
  - Download URLs – double slash
    by vastur
  - map soft – writer: me
    by krzysztof
  - Unicode encoding issue – ne\_10m\_lakes.dbf
    by filter.1

---

Supported by:

© 2009 - 2022. Natural Earth. All rights reserved.


Powered by WordPress

Staff Login »
