# Supplementary material for: Potential patterns of fall armyworm seasonal migration in West Africa modeled with atmospheric trajectory analyses
Source: Pest Manag Sci. 2026 Mar 22;82(7):6509–17. doi: 10.1002/ps.70732 (PMC13240689; doi:10.1002/ps.70732)
Supplement: Supplementary file 2 — Data S2. Supporting Information. [file PS-82-6509-s002.zip › 3_sup 2.zip!/Supplementary Material2/raw data/ne_50m_geography_regions_polys.README.html]

Natural Earth » Blog Archive » Physical Labels - Free vector and raster map data at 1:10m, 1:50m, and 1:110m scales 


Free vector and raster map data at 1:10m, 1:50m, and 1:110m scales

Search for:

- Home
- Features
- Downloads
- Blog
- Issues
- Corrections
- About

---

## Physical Labels

*Area and point labels of major physical features.*  

Download label areas (1.39 MB) version 5.0.0

Download label points (46.54 KB) version 5.0.0

Download elevation points (38.98 KB) version 5.0.0

Download marine areas (667.6 KB) version 5.0.0

**About**

Primarily derived from Patterson’s Physical Map of the World. Polygons defined by international team of volunteers.

**Issues**

**Do not edit this theme!** Instead, edit the 10m version. Once that is edited, select based on scale rank <= 5 and export to make this 50m theme.

The boundaries of physical regions should be taken with a grain of salt. They are roughly accurate to 50m scale, although the number of features included is to the 10m scale.

Mountain peaks have been corrected to their true lat-long location. Spot elevations have also been fixed. Both have been spun off into the new “elevation points” theme. PeakBagger was invaluable in editing this theme.

**Version History**

- 5.0.0
- 4.1.0
- 4.0.0
- 2.0.0
- 1.3.0
- 1.1.0
- 0.8.0

The master changelog is available on Github »

**Feature Lists**

**Area features**

||  |  |  |  |
| --- | --- | --- | --- |
| **Scale Rank** | **Feature Class** | **Name** | **Alternate name** |
| 0 | continent | AFRICA |  |
| 0 | continent | AUSTRALIA |  |
| 0 | continent | SOUTH AMERICA |  |
| 0 | continent | EUROPE |  |
| 0 | continent | ASIA |  |
| 0 | continent | ASIA |  |
| 0 | continent | NORTH AMERICA |  |
| 0 | continent | NORTH AMERICA |  |
| 0 | island group | POLYNESIA |  |
| 0 | island group | POLYNESIA |  |
| 0 | island group | POLYNESIA |  |
| 0 | island group | MICRONESIA |  |
| 0 | island group | MELANESIA |  |
| 1 | geoarea | SIBERIA |  |
| 1 | geoarea | ALASKA |  |
| 1 | ismus | CENTRAL AMERICA |  |
| 1 | plateau | PLATEAU OF TIBET |  |
| 1 | plateau | PENÍNSULA IBÉRICA |  |
| 1 | plateau | WESTERN PLATEAU |  |
| 1 | desert | SAHARA |  |
| 1 | desert | GOBI DESERT |  |
| 1 | penn/cape | ARABIAN PENINSULA |  |
| 1 | penn/cape | INDOCHINA PENINSULA |  |
| 1 | penn/cape | SCANDINAVIA |  |
| 1 | penn/cape | INDIA |  |
| 1 | basin | AMAZON BASIN |  |
| 1 | basin | CONGO BASIN |  |
| 1 | plain | GREAT PLAINS |  |
| 1 | plain | NORTHERN EUROPEAN PLAIN |  |
| 1 | plain | KAZAKH STEPPE |  |
| 1 | plain | NORTH CHINA PLAIN |  |
| 1 | range/mtn | ROCKY MOUNTAINS |  |
| 1 | range/mtn | ANDES |  |
| 1 | range/mtn | HIMALAYAS |  |
| 1 | range/mtn | CAUCASUS MTS. |  |
| 1 | range/mtn | URAL MOUNTAINS |  |
| 1 | range/mtn | TIAN SHAN |  |
| 1 | range/mtn | ALPS |  |
| 1 | range/mtn | GREAT DIVIDING RANGE |  |
| 1 | island group | BRITISH ISLES |  |
| 1 | island group | ARCTIC ARCHIPELAGO |  |
| 1 | island group | NEW ZEALAND |  |
| 1 | island group | JAPAN |  |
| 1 | island group | PHILIPPINES |  |
| 1 | island group | GREATER SUNDA ISLANDS |  |
| 1 | island group | Hawai‘i |  |
| 1 | island group | NEW BRITAIN |  |
| 1 | island group | CAROLINE ISLANDS |  |
| 1 | island group | LINE ISLANDS |  |
| 1 | island group | HAWAIIAN ISLANDS |  |
| 1 | island group | KIRIBATI |  |
| 1 | island group | KIRIBATI |  |
| 1 | tundra | CANADIAN SHIELD |  |
| 1 | tundra | CANADIAN SHIELD |  |
| 1 | island | MADAGASCAR |  |
| 1 | island | GREENLAND |  |
| 2 | geoarea | PATAGONIA |  |
| 2 | geoarea | ANATOLIA |  |
| 2 | foothills | SELVAS |  |
| 2 | tundra | Bol’shezemel’skaya Tundra |  |
| 2 | desert | KALAHARI DESERT |  |
| 2 | desert | CAATINGAS |  |
| 2 | desert | RUB’ AL KHALI |  |
| 2 | desert | PUNJAB |  |
| 2 | desert | BETPAQDALA DESERT |  |
| 2 | penn/cape | BALKAN PEN. |  |
| 2 | penn/cape | SOMALI PENINSULA |  |
| 2 | valley | Fergana Valley |  |
| 2 | plateau | PLANALTO DO MATO GROSSO |  |
| 2 | plateau | DECCAN PLATEAU |  |
| 2 | plateau | MONGOLIAN PLATEAU |  |
| 2 | plateau | CENTRAL SIBERIAN PLATEAU |  |
| 2 | plateau | BRAZILIAN HIGHLANDS |  |
| 2 | plain | GANGES PLAIN |  |
| 2 | plain | PAMPAS |  |
| 2 | plain | LLANOS |  |
| 2 | plain | GRAN CHACO |  |
| 2 | plain | CENTRAL LOWLAND |  |
| 2 | plain | COASTAL PLAIN |  |
| 2 | plain | PONTIC STEPPE |  |
| 2 | plain | SAHEL |  |
| 2 | plain | TURAN LOWLAND |  |
| 2 | plain | MANCHURIAN PLAIN |  |
| 2 | range/mtn | ALASKA RANGE |  |
| 2 | range/mtn | CASCADE RANGE |  |
| 2 | range/mtn | COAST MOUNTAINS |  |
| 2 | range/mtn | BROOKS RANGE |  |
| 2 | range/mtn | APPALACHIAN MTS. |  |
| 2 | range/mtn | APPALACHIAN MTS. |  |
| 2 | range/mtn | ATLAS MOUNTAINS |  |
| 2 | range/mtn | KUNLUN MOUNTAINS |  |
| 2 | range/mtn | KARAKORAM RA. |  |
| 2 | range/mtn | HINDU KUSH |  |
| 2 | range/mtn | PAMIRS |  |
| 2 | range/mtn | Siwalik Hills |  |
| 2 | range/mtn | CHAÎNE ANNAMITIQUE |  |
| 2 | range/mtn | STANOVOY RANGE |  |
| 2 | range/mtn | GREATER KHINGAN RANGE |  |
| 2 | range/mtn | ALTAY MOUNTAINS |  |
| 2 | range/mtn | ZAGROS MOUNTAINS |  |
| 2 | range/mtn | ETHIOPIAN HIGHLANDS |  |
| 2 | basin | GREAT ARTESIAN BASIN |  |
| 2 | basin | GREAT BASIN |  |
| 2 | penn/cape | MALAY PENINSULA |  |
| 2 | penn/cape | KAMCHATKA PENINSULA |  |
| 2 | penn/cape | YAMAL PENINSULA |  |
| 2 | penn/cape | TAYMYR PENINSULA |  |
| 2 | island group | QUEEN ELIZABETH ISLANDS |  |
| 2 | island group | WEST INDIES |  |
| 2 | island group | ALEUTIAN ISLANDS |  |
| 2 | island group | MOLUCCAS |  |
| 2 | island group | NICOBAR ISLANDS |  |
| 2 | island group | ANDAMAN ISLANDS |  |
| 2 | island | ICELAND |  |
| 2 | island | GREAT BRITAIN |  |
| 2 | island | IRELAND |  |
| 2 | island | HONSHÜ |  |
| 2 | island | SUMATRA |  |
| 2 | island | BORNEO |  |
| 2 | island | NEW GUINEA |  |
| 2 | island | TASMANIA |  |
| 2 | island | NORTH ISLAND |  |
| 2 | island | SOUTH ISLAND |  |
| 2 | island | SRI LANKA |  |
| 3 | geoarea | Mesopotamia |  |
| 3 | geoarea | KNUD RASMUSSEN LAND |  |
| 3 | geoarea | SUDD |  |
| 3 | lowland | CASPIAN DEPRESSION |  |
| 3 | lowland | WESTERN SIBERIAN PLAIN |  |
| 3 | lowland | NORTH SIBERIAN LOWLAND |  |
| 3 | wetlands | PANTANAL |  |
| 3 | foothills | YUNGAS |  |
| 3 | foothills | PIEDMONT |  |
| 3 | ismus | ISTHMUS OF KRA |  |
| 3 | basin | TARIM BASIN |  |
| 3 | basin | SICHUAN BASIN |  |
| 3 | basin | MURRAY-DARLING BASIN |  |
| 3 | ismus | Is. de Panamá |  |
| 3 | ismus | Istmo de Tehuantepec |  |
| 3 | desert | DESIERTO DE ATACAMA |  |
| 3 | desert | QIZILQUM DESERT |  |
| 3 | desert | GARAGUM DESERT |  |
| 3 | desert | SYRIAN DESERT |  |
| 3 | desert | THAR DESERT |  |
| 3 | desert | GREAT VICTORIA DESERT |  |
| 3 | desert | LIBYAN DESERT |  |
| 3 | desert | GREAT SANDY DESERT |  |
| 3 | desert | NUBIAN DESERT |  |
| 3 | desert | WESTERN DESERT |  |
| 3 | desert | NAMIB DESERT |  |
| 3 | plain | CHACO AUSTRAL |  |
| 3 | plain | CHACO BOREAL |  |
| 3 | plain | NORTH SLOPE |  |
| 3 | plain | NULLARBOR PLAIN |  |
| 3 | tundra | BARREN GROUNDS |  |
| 3 | plateau | PLANALTO CENTRAL |  |
| 3 | plateau | GUIANA SHIELD |  |
| 3 | plateau | ALTIPLANO |  |
| 3 | plateau | YUKON PLATEAU |  |
| 3 | plateau | CUMBERLAND PLAT. |  |
| 3 | plateau | ALLEGHENY PLAT. |  |
| 3 | plateau | ALTI-PLANICIE MEXICANA |  |
| 3 | plateau | COLUMBIA PLAT. |  |
| 3 | plateau | COLORADO PLATEAU |  |
| 3 | plateau | SHAN PLATEAU |  |
| 3 | plateau | Khorat Plat. |  |
| 3 | plateau | YUNGUI PLATEAU |  |
| 3 | plateau | PUTORANA PLATEAU |  |
| 3 | plateau | Aldan Upland |  |
| 3 | plateau | CENTRAL RUSSIAN UPLAND |  |
| 3 | plateau | KATANGA PLATEAU |  |
| 3 | penn/cape | Shandong Pen. |  |
| 3 | penn/cape | Península Valdés |  |
| 3 | penn/cape | FLORIDA |  |
| 3 | penn/cape | ALASKA PENINSULA |  |
| 3 | penn/cape | PEN. DE YUCATÁN |  |
| 3 | penn/cape | BAJA CALIFORNIA |  |
| 3 | penn/cape | Cà Mau Point |  |
| 3 | penn/cape | KOLA PENINSULA |  |
| 3 | penn/cape | CRIMEA |  |
| 3 | penn/cape | ARNHEM LAND |  |
| 3 | penn/cape | CAPE YORK PEN. |  |
| 3 | valley | GREAT RIFT VALLEY |  |
| 3 | valley | GREAT RIFT VALLEY |  |
| 3 | delta | Mekong Delta |  |
| 3 | delta | Amazon Delta |  |
| 3 | delta | Danube Delta |  |
| 3 | delta | Volga Delta |  |
| 3 | delta | Niger Delta |  |
| 3 | delta | Mississippi Delta |  |
| 3 | range/mtn | CORDILLERA ORIENTAL |  |
| 3 | range/mtn | CORD. CENTRAL |  |
| 3 | range/mtn | CORD. OCCIDENTAL |  |
| 3 | range/mtn | CORD. CENTRAL |  |
| 3 | range/mtn | CORDILLERA ORIENTAL |  |
| 3 | range/mtn | CORDILLERA REAL |  |
| 3 | range/mtn | CORDILLERA OCCIDENTAL |  |
| 3 | range/mtn | MACKENZIE MTS. |  |
| 3 | range/mtn | CHUGACH MTS. |  |
| 3 | range/mtn | SIERRA MADRE OCCIDENTAL |  |
| 3 | range/mtn | SIERRA NEVADA |  |
| 3 | range/mtn | SIERRA MADRE ORIENTAL |  |
| 3 | range/mtn | COAST RANGES |  |
| 3 | range/mtn | Sierra Madre del Sur |  |
| 3 | range/mtn | PYRENEES |  |
| 3 | range/mtn | APPENNINI |  |
| 3 | range/mtn | CARPATHIAN MOUNTAINS |  |
| 3 | range/mtn | KJØLEN MOUNTAINS |  |
| 3 | range/mtn | ELBURZ MTS. |  |
| 3 | range/mtn | ASIR MTS. |  |
| 3 | range/mtn | HEJAZ MTS. |  |
| 3 | range/mtn | ALTUN MTS. |  |
| 3 | range/mtn | Tarbagatay Ra. |  |
| 3 | range/mtn | Wuyi Mts. |  |
| 3 | range/mtn | Vindhya Range |  |
| 3 | range/mtn | EASTERN GHATS |  |
| 3 | range/mtn | WESTERN GHATS |  |
| 3 | range/mtn | Nan Ling Mts. |  |
| 3 | range/mtn | Dabie Mts. |  |
| 3 | range/mtn | YABLONOVYY RANGE |  |
| 3 | range/mtn | EASTERN SAYAN MTS. |  |
| 3 | range/mtn | QUILIAN MOUNTAINS |  |
| 3 | range/mtn | Central Highlands |  |
| 3 | range/mtn | VERKHOYANSK RANGE |  |
| 3 | range/mtn | SIKHOTE-ALIN’ RANGE |  |
| 3 | range/mtn | Lesser Khingan Range |  |
| 3 | range/mtn | SOUTHERN ALPS |  |
| 3 | range/mtn | MITUMBA MTS. |  |
| 3 | range/mtn | AÏR MTS. |  |
| 3 | range/mtn | AHAGGAR MTS. |  |
| 3 | range/mtn | TIBESTI MTS. |  |
| 3 | range/mtn | CRYSTAL MOUNTAINS |  |
| 3 | range/mtn | DRAKENSBERG |  |
| 3 | range/mtn | HAUT ATLAS |  |
| 3 | range/mtn | ATLAS SAHARIEN |  |
| 3 | island group | AÇORES |  |
| 3 | island group | PARRY ISLANDS |  |
| 3 | island group | LESSER ANTILLES |  |
| 3 | island group | BAHAMA ISLANDS |  |
| 3 | island group | GREATER ANTILLES |  |
| 3 | island group | NEW SIBERIAN ISLANDS |  |
| 3 | island group | ALEUTIAN ISLANDS |  |
| 3 | island group | Spratly Islands |  |
| 3 | island group | LESSER SUNDA ISLANDS |  |
| 3 | island group | Balearic Is. |  |
| 3 | island group | NOVAYA ZEMLYA | New Land |
| 3 | island group | FRANZ JOSEF LAND |  |
| 3 | island group | SVALBARD |  |
| 3 | island group | FALKLAND IS. | IS. MALVINAS |
| 3 | island group | SEVERNAYA ZEMLYA | North Land |
| 3 | island group | Myeik Arch. |  |
| 3 | island group | SEYCHELLES |  |
| 3 | island group | ARCHIPEL DES COMORES |  |
| 3 | island group | MASCARENE ISLANDS |  |
| 3 | island group | Viti Levu |  |
| 3 | island group | Maui |  |
| 3 | island group | Tahiti |  |
| 3 | island group | Isabela |  |
| 3 | island group | Guadalcanal |  |
| 3 | island group | Bougainville |  |
| 3 | island group | Bismarck Archipelago |  |
| 3 | island group | ARCHIPEL DES TUAMOTU |  |
| 3 | island group | RYÜKYÜ ISANDS |  |
| 3 | island group | MARSHALL ISANDS |  |
| 3 | island group | SOLOMON ISANDS |  |
| 3 | island group | Espiritu Santo |  |
| 3 | island | Sicilia |  |
| 3 | island | Corse |  |
| 3 | island | Sardegna |  |
| 3 | island | SAKHALIN |  |
| 3 | island | HOKKAIDÖ |  |
| 3 | island | SHIKOKU |  |
| 3 | island | KYÜSHÜ |  |
| 3 | island | TAIWAN |  |
| 3 | island | LUZON |  |
| 3 | island | MINDANAO |  |
| 3 | island | JAVA |  |
| 3 | island | SULAWESI |  |
| 3 | island | ISLAND OF NEWFOUNDLAND |  |
| 3 | island | BAFFIN ISLAND |  |
| 3 | island | ELLESMERE ISLAND |  |
| 3 | island | LABRADOR |  |
| 3 | island | VICTORIA ISLAND |  |
| 3 | island | Vancouver Island |  |
| 3 | island | Nova Scotia |  |
| 3 | island | TIERRA DEL FUEGO |  |
| 3 | island | N. Andaman |  |
| 3 | island | Middle Andaman |  |
| 3 | island | S. Andaman |  |
| 3 | island | Great Nicobar |  |
| 3 | island | Bol’shoy Begichev I. |  |
| 4 | wetlands | Entre Rios |  |
| 4 | valley | Central Valley |  |
| 4 | valley | Valle Longitudinal |  |
| 4 | gorge | Tsangpo Gorge |  |
| 4 | gorge | Yangtze Gorges |  |
| 4 | gorge | Grand Canyon |  |
| 4 | geoarea | JUTLAND |  |
| 4 | geoarea | JUTLAND |  |
| 4 | geoarea | LAPLAND |  |
| 4 | geoarea | BALUCHISTAN |  |
| 4 | geoarea | Hexi Corridor |  |
| 4 | geoarea | Riverina |  |
| 4 | geoarea | Banaadir Coast |  |
| 4 | geoarea | Ogaden |  |
| 4 | lowland | VYCHEGDA LOWLAND |  |
| 4 | lowland | KOLYMA LOWLAND |  |
| 4 | basin | Qaidam Basin |  |
| 4 | basin | Junggar Basin |  |
| 4 | plateau | Loess Plateau |  |
| 4 | plateau | STANOVOY UPLAND |  |
| 4 | plateau | Ustyurt Plateau |  |
| 4 | plateau | KAZAKH UPLAND |  |
| 4 | plateau | KIMBERLEY PLATEAU |  |
| 4 | plateau | Superior Upland |  |
| 4 | plateau | FRASER PLATEAU |  |
| 4 | plateau | Edwards Plateau |  |
| 4 | plateau | Ozark Plateau |  |
| 4 | plateau | Campos Sertão |  |
| 4 | plateau | Bié Plateau |  |
| 4 | plateau | Huíla Plateau |  |
| 4 | plateau | Adamawa Plateau |  |
| 4 | plain | LES LAURENTIDES |  |
| 4 | plain | Yukon Flats |  |
| 4 | plain | Iwembere Steppe |  |
| 4 | penn/cape | Leizhou Pen. |  |
| 4 | penn/cape | Kathiawar Pen. |  |
| 4 | penn/cape | Liaodong Pen. |  |
| 4 | penn/cape | Cape Chelyuskin |  |
| 4 | penn/cape | GYDA PENINSULA |  |
| 4 | penn/cape | KOREA |  |
| 4 | penn/cape | Pén. de Gaspé |  |
| 4 | penn/cape | PÉNINSULE D’UNGAVA |  |
| 4 | penn/cape | Melville Peninsula |  |
| 4 | penn/cape | Cumberland Peninsula |  |
| 4 | penn/cape | CHUKCHI PENINSULA |  |
| 4 | coast | Dalmatia |  |
| 4 | coast | Costa de los Mosquitos |  |
| 4 | desert | LUT DESERT |  |
| 4 | desert | TAKLIMAKAN DESERT |  |
| 4 | desert | Mu Us Desert | Ordos |
| 4 | desert | Gibson Desert |  |
| 4 | desert | Great Salt Lake Desert |  |
| 4 | desert | SONORAN DESERT |  |
| 4 | desert | CHIHUAHUAN DESERT |  |
| 4 | desert | El Djouf |  |
| 4 | desert | ‘Erg Chech |  |
| 4 | desert | Grand Erg Occidental |  |
| 4 | desert | Grand Erg Oriental |  |
| 4 | desert | Sarïr Kalanshiyu ar Ramli al Kabi | Sea of Sand |
| 4 | desert | Tassili-n-Ajjer |  |
| 4 | desert | Tassili oua-n-Ahaggar |  |
| 4 | desert | Ténéré |  |
| 4 | desert | Hamada du Drâa |  |
| 4 | desert | Idhän Murzuq |  |
| 4 | range/mtn | Hadhramaut |  |
| 4 | range/mtn | Sierra Morena |  |
| 4 | range/mtn | S. Nevada |  |
| 4 | range/mtn | Cord. Cantábrica |  |
| 4 | range/mtn | Dinaric Alps |  |
| 4 | range/mtn | Balkan Mts. |  |
| 4 | range/mtn | KUH RUD MOUNTAINS |  |
| 4 | range/mtn | PONTIC MOUNTAINS |  |
| 4 | range/mtn | Lesser Caucasus |  |
| 4 | range/mtn | Khasi Hills |  |
| 4 | range/mtn | Alataw Mts. |  |
| 4 | range/mtn | Borohoro Mts. |  |
| 4 | range/mtn | Salt Ra. |  |
| 4 | range/mtn | Satpura Range |  |
| 4 | range/mtn | Southern Ghats |  |
| 4 | range/mtn | Arakan Yoma |  |
| 4 | range/mtn | Naga Hills |  |
| 4 | range/mtn | Dângrêk Ra. |  |
| 4 | range/mtn | Dalou Mts. |  |
| 4 | range/mtn | Qinling Mountains |  |
| 4 | range/mtn | Lüliang Mts. |  |
| 4 | range/mtn | Taihang Mts. |  |
| 4 | range/mtn | Tannu-Ola Ra. |  |
| 4 | range/mtn | Abakanskiy Ra. |  |
| 4 | range/mtn | Western Sayan Mts. |  |
| 4 | range/mtn | Yin Mts. |  |
| 4 | range/mtn | Chin Hills |  |
| 4 | range/mtn | CHERSKIY RANGE |  |
| 4 | range/mtn | KOLYMA RANGE |  |
| 4 | range/mtn | Koryak Range |  |
| 4 | range/mtn | SREDINNYY RANGE |  |
| 4 | range/mtn | Hamgyong Mts. |  |
| 4 | range/mtn | Hangayn Mts. |  |
| 4 | range/mtn | NEW GUINEA HIGHLANDS |  |
| 4 | range/mtn | TORNGAT MTS. |  |
| 4 | range/mtn | COLUMBIA MTS. |  |
| 4 | range/mtn | SELWYN MTS. |  |
| 4 | range/mtn | Sierra Chiapas |  |
| 4 | range/mtn | GUIANA HIGHLANDS |  |
| 4 | range/mtn | Serra da Mantiqueira |  |
| 4 | range/mtn | Serra Geral |  |
| 4 | range/mtn | Serra do Mar |  |
| 4 | range/mtn | Muchinga Mts. |  |
| 4 | range/mtn | Anti Atlas |  |
| 4 | coast | Coromandel Coast |  |
| 4 | coast | Malabar Coast |  |
| 4 | delta | Ganges Delta |  |
| 4 | delta | Irrawaddy Delta |  |
| 4 | delta | Indus Delta |  |
| 4 | delta | Lena Delta |  |
| 4 | delta | Nile Delta |  |
| 4 | delta | Zambezi Delta |  |
| 4 | island group | Virgin Is. |  |
| 4 | island group | Channel Islands |  |
| 4 | island group | Alexander Archipelago |  |
| 4 | island group | Queen Charlotte Islands |  |
| 4 | island group | Islas Revillagigedo |  |
| 4 | island group | AÇORES |  |
| 4 | island group | ISLAS CANARIAS |  |
| 4 | island group | ILHAS DE CABO VERDE |  |
| 4 | island group | Sulu Archipelago |  |
| 4 | island group | Amami |  |
| 4 | island group | Okinawa |  |
| 4 | island group | Paramushir |  |
| 4 | island group | Guam |  |
| 4 | island group | Vanua Levu |  |
| 4 | island group | Kaua‘i |  |
| 4 | island group | O‘ahu |  |
| 4 | island group | Kiritimati | Christmas I. |
| 4 | island group | Nuku Hiva |  |
| 4 | island group | Hiva Oa |  |
| 4 | island group | Raiatea |  |
| 4 | island group | Savai‘i |  |
| 4 | island group | Santa Cruz |  |
| 4 | island group | Malakula |  |
| 4 | island group | Santa Isabel |  |
| 4 | island group | San Cristobal |  |
| 4 | island group | New Ireland |  |
| 4 | island group | NORTHERN COOK ISLANDS |  |
| 4 | island group | SOUTHERN COOK ISLANDS |  |
| 4 | island group | NAMPÖ-SHOTÖ |  |
| 4 | island group | RALIK CHAIN |  |
| 4 | island group | RATAK CHAIN |  |
| 4 | island group | FIJI |  |
| 4 | island group | TONGA ISLANDS |  |
| 4 | island group | FIJI |  |
| 4 | island group | KURIL ISANDS |  |
| 4 | island | CUBA |  |
| 4 | island | HISPANIOLA |  |
| 4 | island | Cape Breton Island |  |
| 4 | island | Prince Edward I. |  |
| 4 | island | Trinidad |  |
| 4 | island | Puerto Rico |  |
| 4 | island | Jamaica |  |
| 4 | island | BANKS ISLAND |  |
| 4 | island | Cyprus |  |
| 4 | island | Mindoro |  |
| 4 | island | Panay |  |
| 4 | island | Samar |  |
| 4 | island | Leyte |  |
| 4 | island | Negros |  |
| 4 | island | Bohol |  |
| 4 | island | Palawan |  |
| 4 | island | TIMOR |  |
| 4 | island | Halmahera |  |
| 4 | island | Ceram |  |
| 4 | island | Isla de Chiloé |  |
| 4 | island | Shmidta I. |  |
| 4 | island | Arctic Institute I. |  |
| 4 | island | October Revolution I. |  |
| 4 | island | Bol’shevik I. |  |
| 4 | island | Komsomolets I. |  |
| 4 | island | HAINAN |  |
| 5 | geoarea | Nyeboe Land |  |
| 5 | geoarea | Peary Land |  |
| 5 | geoarea | Crown Prince Christian Land |  |
| 5 | geoarea | King Frederik VIII Land |  |
| 5 | geoarea | King Wilhelm Land |  |
| 5 | geoarea | King Christian X Land |  |
| 5 | geoarea | King Christian IX Land |  |
| 5 | geoarea | Jameson Land |  |
| 5 | geoarea | Karelia |  |
| 5 | geoarea | Finnish Lake Region |  |
| 5 | geoarea | Bretagne |  |
| 5 | geoarea | Normandie |  |
| 5 | geoarea | Dampier Land |  |
| 5 | geoarea | El Gezira |  |
| 5 | wetlands | Sundarbans |  |
| 5 | valley | Vale of Kashmir |  |
| 5 | depression | Bodélé Depression |  |
| 5 | depression | Aoukâr |  |
| 5 | coast | King Frederik VI Coast |  |
| 5 | coast | Riviera |  |
| 5 | coast | Eighty Mile Beach |  |
| 5 | coast | Gold Coast |  |
| 5 | coast | Ivory Coast |  |
| 5 | coast | Slave Coast |  |
| 5 | coast | Grain Coast |  |
| 5 | coast | Levant Coast |  |
| 5 | coast | Fiordland |  |
| 5 | plain | Llano Estacado |  |
| 5 | plain | Baltic Plains |  |
| 5 | plain | Alföld |  |
| 5 | plain | Hasa Plain |  |
| 5 | plain | Tihamah |  |
| 5 | plain | Darling Downs |  |
| 5 | plain | Jabal Nafüsah |  |
| 5 | plain | Masai Steppe |  |
| 5 | plain | Serengeti Plains |  |
| 5 | desert | Big Bend |  |
| 5 | desert | An Nafud Desert |  |
| 5 | desert | Kavir Desert |  |
| 5 | desert | Margo Desert |  |
| 5 | desert | Rigestan |  |
| 5 | desert | Tanami Desert |  |
| 5 | desert | Simpson Desert |  |
| 5 | desert | Strzelecki Desert |  |
| 5 | desert | ‘Erg Iguidi |  |
| 5 | desert | Hamada de Tinrhert |  |
| 5 | desert | Akchâr |  |
| 5 | desert | Trarza |  |
| 5 | desert | Eastern Desert |  |
| 5 | desert | Chalbi Desert |  |
| 5 | desert | Negev Desert |  |
| 5 | desert | Danakil |  |
| 5 | desert | Wahiba Sands |  |
| 5 | desert | Salar de Uyuni |  |
| 5 | plateau | Northern Karroo |  |
| 5 | plateau | Planalto da Borborema |  |
| 5 | plateau | Chapada do Araripe |  |
| 5 | plateau | Finnmarks-vidda |  |
| 5 | plateau | Massif Central |  |
| 5 | plateau | Azov Upland |  |
| 5 | plateau | Dnieper Upland |  |
| 5 | plateau | Volga Upland |  |
| 5 | plateau | Stavropol’ Plateau |  |
| 5 | plateau | Chota Nägpur Plateau |  |
| 5 | plateau | Okhotsk-Kolyma Upland |  |
| 5 | plateau | Barkly Tableland |  |
| 5 | plateau | Atherton Tableland |  |
| 5 | plateau | Buckland Tableland |  |
| 5 | plateau | Libyan Plateau |  |
| 5 | plateau | Jabal al Abyad Plateau |  |
| 5 | plateau | Jilf al Kabir Plateau |  |
| 5 | plateau | Plat. du Tademaït |  |
| 5 | plateau | Fouta Djallon |  |
| 5 | plateau | Tagant |  |
| 5 | plateau | Great Karroo |  |
| 5 | penn/cape | Pen. de Azuero |  |
| 5 | penn/cape | Pen. de la Guajira |  |
| 5 | penn/cape | Seward Peninsula |  |
| 5 | penn/cape | Kenai Peninsula |  |
| 5 | penn/cape | Meta Incognita Pen. |  |
| 5 | penn/cape | Foxe Pen. |  |
| 5 | penn/cape | Boothia Peninsula |  |
| 5 | penn/cape | Koni Pen. |  |
| 5 | penn/cape | P’yagina Pen. |  |
| 5 | penn/cape | Taygonos Pen. |  |
| 5 | penn/cape | Datu Pen. |  |
| 5 | penn/cape | Wilsons Promontory |  |
| 5 | penn/cape | Banks Pen. |  |
| 5 | penn/cape | Noto Pen. |  |
| 5 | penn/cape | Cobourg Pen. |  |
| 5 | penn/cape | Musandam Peninsula |  |
| 5 | penn/cape | Absheron Pen. |  |
| 5 | penn/cape | Kanin Pen. |  |
| 5 | penn/cape | Península de Taitao |  |
| 5 | range/mtn | S. de Pakaraima |  |
| 5 | range/mtn | Serra do Espinhaço |  |
| 5 | range/mtn | S. dos Gradaús |  |
| 5 | range/mtn | S. Dourada |  |
| 5 | range/mtn | S. do Cachimbo |  |
| 5 | range/mtn | Serra de Curupira |  |
| 5 | range/mtn | Serra Acaraí |  |
| 5 | range/mtn | Serra da Ibiapaba |  |
| 5 | range/mtn | S. do Piaui |  |
| 5 | range/mtn | Wilhelmina Gebergte |  |
| 5 | range/mtn | Tumuc-Humac Mts. |  |
| 5 | range/mtn | Cuchilla Grande |  |
| 5 | range/mtn | Cord. de Mérida |  |
| 5 | range/mtn | Mogollon Rim |  |
| 5 | range/mtn | Black Hills |  |
| 5 | range/mtn | Salmon River Mts. |  |
| 5 | range/mtn | Ouachita Mts. |  |
| 5 | range/mtn | Klamath Mts. |  |
| 5 | range/mtn | Blue Ridge |  |
| 5 | range/mtn | Adirondack Mts. |  |
| 5 | range/mtn | Wrangell Mts. |  |
| 5 | range/mtn | Ogilvie Mts. |  |
| 5 | range/mtn | Cassiar Mts. |  |
| 5 | range/mtn | Caribou Mts. |  |
| 5 | range/mtn | Birch Mts. |  |
| 5 | range/mtn | Vatnajökull |  |
| 5 | range/mtn | Cambrian Mts. |  |
| 5 | range/mtn | Grampian Mts. |  |
| 5 | range/mtn | Jura |  |
| 5 | range/mtn | Ardennes |  |
| 5 | range/mtn | Erzgebirge |  |
| 5 | range/mtn | Sudetes Mts. |  |
| 5 | range/mtn | Vosges |  |
| 5 | range/mtn | Harz |  |
| 5 | range/mtn | Calabria |  |
| 5 | range/mtn | Pindus Mts. |  |
| 5 | range/mtn | Tatra Mts. |  |
| 5 | range/mtn | Transylvanian Alps |  |
| 5 | range/mtn | Böhmerwald |  |
| 5 | range/mtn | Taurus Mts. |  |
| 5 | range/mtn | Tuwayq Mts. |  |
| 5 | range/mtn | Al Hajar Mts. |  |
| 5 | range/mtn | Paropamisus Ra. |  |
| 5 | range/mtn | Kopet Dag Mts . |  |
| 5 | range/mtn | Lebanon Mts. |  |
| 5 | range/mtn | Kirthar Ra. |  |
| 5 | range/mtn | Central Makran |  |
| 5 | range/mtn | Sulaiman Ra. |  |
| 5 | range/mtn | Arvalli Ra. |  |
| 5 | range/mtn | Karatau Range |  |
| 5 | range/mtn | Central Bruhui Range |  |
| 5 | range/mtn | Momskiy Mts. |  |
| 5 | range/mtn | Dzhugdzhur Range |  |
| 5 | range/mtn | Suntar-Khayata Range |  |
| 5 | range/mtn | Chukchi Range |  |
| 5 | range/mtn | Yam Alin’ Range |  |
| 5 | range/mtn | Bureinskiy Ra. |  |
| 5 | range/mtn | Hentiyn Mts. |  |
| 5 | range/mtn | Barisan Mountains |  |
| 5 | range/mtn | Maoke Mts. |  |
| 5 | range/mtn | Owen Stanley Ra. |  |
| 5 | range/mtn | Hamersley Ra. |  |
| 5 | range/mtn | Darling Range |  |
| 5 | range/mtn | MacDonnell Ranges |  |
| 5 | range/mtn | Selwyn Ra. |  |
| 5 | range/mtn | Gregory Ra. |  |
| 5 | range/mtn | Blue Mts. |  |
| 5 | range/mtn | Flinders Ranges |  |
| 5 | range/mtn | Australian Alps |  |
| 5 | range/mtn | Inyanga Mts. |  |
| 5 | range/mtn | Atlas Tellien |  |
| 5 | range/mtn | Er Rif |  |
| 5 | range/mtn | Al Jabal al Akhdar |  |
| 5 | range/mtn | Ahmar Mts. |  |
| 5 | range/mtn | Marrah Mts. |  |
| 5 | range/mtn | Guinea Highlands |  |
| 5 | range/mtn | Groot Karasberge |  |
| 5 | range/mtn | Ogo Mts. |  |
| 5 | range/mtn | Chaîne des Mongos |  |
| 5 | range/mtn | Hardanger-vidda |  |
| 5 | range/mtn | Bitterroot Range |  |
| 5 | range/mtn | Front Range |  |
| 5 | range/mtn | Maya Mts. |  |
| 5 | coast | Skeleton Coast |  |
| 5 | coast | Wild Coast |  |
| 5 | pen/cape | Cape Zhelaniya |  |
| 5 | pen/cape | Taz Pen. |  |
| 5 | island group | Belcher Islands |  |
| 5 | island group | Frisian Is. |  |
| 5 | island group | Cyclades |  |
| 5 | island group | Shetland Is. |  |
| 5 | island group | Føroyar | Faroe Is. |
| 5 | island group | Orkney Is. |  |
| 5 | island group | Outer Hebrides |  |
| 5 | island group | Ionian Is. |  |
| 5 | island group | Auckland Islands |  |
| 5 | island group | Auckland Islands |  |
| 5 | island group | Lyakhov Islands |  |
| 5 | island group | Lyakhov Islands |  |
| 5 | island group | Bear Is. |  |
| 5 | island group | Commander Islands |  |
| 5 | island group | Shantar Islands |  |
| 5 | island group | Oki Is. |  |
| 5 | island group | Ösumi Is. |  |
| 5 | island group | Babuyan Is. |  |
| 5 | island group | Calamian Group |  |
| 5 | island group | Batu Is. |  |
| 5 | island group | Batu Is. |  |
| 5 | island group | Riau Arch. |  |
| 5 | island group | Talaud Is. |  |
| 5 | island group | Talaud Is. |  |
| 5 | island group | Talaud Is. |  |
| 5 | island group | Sangihe Islands |  |
| 5 | island group | Tanimbar Islands |  |
| 5 | island group | Aru Islands |  |
| 5 | island group | Whitsunday Group |  |
| 5 | island group | Bonaparte |  |
| 5 | island group | LACCADIVE IS. |  |
| 5 | island group | SAMOA IS. |  |
| 5 | island group | Iturup |  |
| 5 | island group | Urup |  |
| 5 | island group | ARCHIPIÉLAGO DE COLÓN | GALAPAGOS IS. |
| 5 | island group | ÎLES MARQUESAS |  |
| 5 | island group | ÎLES DE LA SOCIÉTÉ |  |
| 5 | island group | ÎLES GAMBIER |  |
| 5 | island group | MARIANA ISANDS |  |
| 5 | island group | TUVALU |  |
| 5 | island group | NOUVELLE-CALÉDONIE |  |
| 5 | island group | VANUATU |  |
| 5 | island group | ÎLES TUBUAÏ |  |
| 5 | island group | PHOENIX ISLANDS |  |
| 5 | island group | Is. Revillagigedo |  |
| 5 | island group | IZU-SHOTÖ |  |
| 5 | island group | SANTA CRUZ ISLANDS |  |
| 5 | island group | PALAU IS. |  |
| 5 | island group | Lau Group |  |
| 5 | island | Kodiak Island |  |
| 5 | island | Wrangel I. |  |
| 5 | island | Devon Island |  |
| 5 | island | Axel Heiberg I. |  |
| 5 | island | Prince of Wales I. |  |
| 5 | island | Southampton I. |  |
| 5 | island | Melville Island |  |
| 5 | island | Qeqertarsuaq | Disko I. |
| 5 | island | Grand Bahama |  |
| 5 | island | Long I. |  |
| 5 | island | Madeira |  |
| 5 | island | Gran Canaria |  |
| 5 | island | Tenerife |  |
| 5 | island | Bioko |  |
| 5 | island | Socotra |  |
| 5 | island | Zanzibar I. |  |
| 5 | island | Príncipe |  |
| 5 | island | São Tomé |  |
| 5 | island | Jan Mayen |  |
| 5 | island | Kolguyev I. |  |
| 5 | island | Vaygach I. |  |
| 5 | island | Nordaustlandet |  |
| 5 | island | Spitsbergen |  |
| 5 | island | Edgeøya |  |
| 5 | island | Lesbos |  |
| 5 | island | Bjørnøya | Bear I. |
| 5 | island | Ibiza |  |
| 5 | island | Mallorca |  |
| 5 | island | Menorca |  |
| 5 | island | Malta |  |
| 5 | island | Kvitøya |  |
| 5 | island | Zemlya Georga I. |  |
| 5 | island | Greem Bell I. |  |
| 5 | island | Crete |  |
| 5 | island | Rhodes |  |
| 5 | island | Pelopónnisos |  |
| 5 | island | Vesterålen |  |
| 5 | island | Lofoten |  |
| 5 | island | Zealand |  |
| 5 | island | Gotland |  |
| 5 | island | Åland |  |
| 5 | island | Saaremaa |  |
| 5 | island | Stewart I. |  |
| 5 | island | Simeulue |  |
| 5 | island | Nias |  |
| 5 | island | Siberut |  |
| 5 | island | South Pagai |  |
| 5 | island | Enggano |  |
| 5 | island | Natuna Basar |  |
| 5 | island | Bangka |  |
| 5 | island | Belitung |  |
| 5 | island | Madura I. |  |
| 5 | island | Bali |  |
| 5 | island | Lombok |  |
| 5 | island | Sumbawa |  |
| 5 | island | Sumba |  |
| 5 | island | Flores |  |
| 5 | island | Wetar |  |
| 5 | island | Alor |  |
| 5 | island | Morotai |  |
| 5 | island | Taliabu |  |
| 5 | island | Buru |  |
| 5 | island | Yos Sudarso I. |  |
| 5 | island | Biak |  |
| 5 | island | Melville I. |  |
| 5 | island | Fraser I. |  |
| 5 | island | Kangaroo I. |  |
| 5 | island | Flinders I. |  |
| 5 | island | King I. |  |
| 5 | island | Rowley Shoals |  |
| 5 | island | Kotel’nyy I. |  |
| 5 | island | Novaya Sibir’ I. |  |
| 5 | island | Ayon I. |  |
| 5 | island | Wrangel I. |  |
| 5 | island | Karaginskiy I. |  |
| 5 | island | Mornington I. |  |
| 5 | island | Groote Eylandt |  |
| 5 | island | East Falkland |  |
| 5 | island | West Falkland |  |
| 5 | island | Cheju I. |  |
| 5 | island | Belyy I. |  |
| 5 | island | Ushakova I. |  |
| 5 | island | Vize I. |  |
| 5 | island | Ilha de Marajó |  |
| 5 | island | Niue |  |
| 5 | island | Tongatapu |  |
| 5 | island | Taveuni |  |
| 5 | island | Tutuila |  |
| 5 | island | Upolu |  |
| 5 | island | Ni‘ihau |  |
| 5 | island | Läna‘i |  |
| 5 | island | Ua Pou |  |
| 5 | island | Moorea |  |
| 5 | island | Bora Bora |  |
| 5 | island | Moloka‘i |  |
| 5 | island | Rota |  |
| 5 | island | Saipan |  |
| 5 | island | Kadavu |  |
| 5 | island | Pohnpei |  |
| 5 | island | Tanna |  |
| 5 | island | Éfaté |  |
| 5 | island | Choiseul |  |
| 5 | island | Malaita |  |
| 5 | island | Manus |  |

**Point locations with elevation**

**Scale Rank**

**Feature Class** |  
 **Name** |  
 **Elevation (meters)** |  
 **Comment** |  
 **Alternative Name** |  
  
|  
  |

2

mountain |  
 Vinson Massif |  
 4892 |  
  |

3

mountain |  
 Mt. Takahe |  
 3460 |  
  |

3

mountain |  
 Mt. Sidley |  
 4285 |  
  |

3

mountain |  
 Mt. Fridtjof Nansen |  
 4069 |  
  |

4

mountain |  
 Chimborazo |  
 6267 |  
  |

4

mountain |  
 Pico Bolívar |  
 4980 |  
  |

4

mountain |  
 Klyuchevskaya Sopka |  
 4750 |  
  |

4

mountain |  
 Puncak Jaya |  
 4884 |  
  |

4

mountain |  
 Gunung Kinabalu |  
 4101 |  
  |

5

mountain |  
 Otgon Tenger |  
 4000 |  
  |

5

mountain |  
 Gora Korolenko |  
 1647 |  
  |

5

mountain |  
 Magen Gangri |  
 6282 |  
  |

5

mountain |  
 Taibai Shan |  
 3767 |  
  |

5

mountain |  
 Nanda Devi |  
 7817 |  
  |

5

mountain |  
 Daba Shan |  
 2708 |  
  |

5

mountain |  
 Mazong Shan |  
 2584 |  
  |

5

mountain |  
 Gora Burun-Shibertuy |  
 2519 |  
  |

5

mountain |  
 Skalistyy Golets |  
 2467 |  
  |

5

mountain |  
 Inyaptuk Golets |  
 2641 |  
  |

5

mountain |  
 Grandiozny Pk. |  
 2891 |  
  |

5

mountain |  
 Jebel Toubkal |  
 4165 |  
  |

5

mountain |  
 Thabana Ntlenyana |  
 3482 |  
  |

5

mountain |  
 Mt. Batu |  
 4307 |  
  |

5

mountain |  
 Argun |  
 3418 |  
  |

5

mountain |  
 Kinyeti |  
 3187 |  
  |

5

mountain |  
 Muleta |  
 3405 |  
  |

5

mountain |  
 Ras Dejen |  
 4533 |  
  |

5

mountain |  
 Mt. Elgon |  
 4321 |  
  |

5

mountain |  
 Wheeler Pk. |  
 3982 |  
  |

5

mountain |  
 Borah Pk. |  
 3859 |  
  |

5

mountain |  
 Sacajawea Pk. |  
 2999 |  
  |

5

mountain |  
 Trapper Pk. |  
 3096 |  
  |

5

mountain |  
 Kings Pk. |  
 4123 |  
  |

5

mountain |  
 Mt. Baker |  
 3286 |  
  |

5

mountain |  
 Mt. Elbert |  
 4402 |  
  |

5

mountain |  
 Uncompahgre Pk. |  
 4361 |  
  |

5

mountain |  
 Mt. Mitchell |  
 2037 |  
  |

5

mountain |  
 Clingmans Dome |  
 2025 |  
  |

5

mountain |  
 Spruce Knob |  
 1482 |  
  |

5

mountain |  
 Mt. Rogers |  
 1746 |  
  |

5

depression |  
 Qattara Depression |  
 -133 |  
  |

5

depression |  
 Er Richat Depression |  
 485 |  
  |

5

mountain |  
 Talo |  
 4413 |  
  |

5

mountain |  
 Lesatima |  
 4001 |  
  |

4

mountain |  
 Bogda Feng |  
 5445 |  
  |

4

mountain |  
 Pik Imeni Ismail Samani |  
 7495 |  
  |

4

mountain |  
 Gora Manas |  
 4482 |  
  |

4

mountain |  
 Shah Fuladi |  
 5048 |  
  |

4

mountain |  
 K?h-e Jang Qal’eh |  
 4171 |  
  |

4

mountain |  
 Gora Munku-Sardyk |  
 3491 |  
  |

4

mountain |  
 Wutai Shan |  
 3058 |  
  |

4

mountain |  
 Mt. Kenya |  
 5199 |  
  |

4

mountain |  
 Mt. Stanley |  
 4979 |  
  |

3

mountain |  
 Gongga Shan |  
 7556 |  
  |

3

mountain |  
 Dhaulagiri |  
 8172 |  
  |

3

mountain |  
 Belukha |  
 4506 |  
  |

3

mountain |  
 Nganglong Kangri |  
 6720 |  
  |

3

mountain |  
 Geladandong |  
 6621 |  
  |

3

mountain |  
 Mt. Washington |  
 1917 |  
  |

3

mountain |  
 Mt. Katahdin |  
 1606 |  
  |

3

mountain |  
 Mt. Shasta |  
 4322 |  
  |

3

mountain |  
 Grand Teton |  
 4199 |  
  |

3

mountain |  
 Mt. Hood |  
 3426 |  
  |

2

mountain |  
 Mt. Kilimanjaro |  
 5895 |  
 Highest point in Africa |  
  |

3

mountain |  
 Mt. Blanc |  
 4807 |  
  |

3

depression |  
  |

2

mountain |  
 Mt. Everest |  
 8848 |  
 Worlds highest point |  
  |

4

mountain |  
 Kangtö |  
 7060 |  
  |

2

depression |  
 Death Valley |  
 -86 |  
 Lowest point in N. America |  
  |

2

mountain |  
 Mt. McKinley |  
 6194 |  
 Highest point in N. America |  
  |

4

mountain |  
 Mt. Chamberlin |  
 2749 |  
  |

2

depression |  
 Turpan Depression |  
 -154 |  
  |

2

mountain |  
 Cerro Aconcagua |  
 6959 |  
 Highest point in S. America |  
  |

4

mountain |  
 Cerro Champaquí |  
 2770 |  
  |

2

depression |  
  |

2

depression |  
  |

2

depression |  
  |

2

mountain |  
 Gora Elbrus |  
 5642 |  
 Highest point in Europe |  
 Mount Elbrus |  
  
|  
  |

2

depression |  
  |

2

mountain |  
 Cero Raya |  
 2070 |  
  |

2

pass |  
 Khyber Pass |  
 1070 |  
  |

2

mountain |  
 Mt. Whitney |  
 4421 |  
  |

2

mountain |  
 K2 |  
 8611 |  
  |

2

mountain |  
 Muztag Feng |  
 6973 |  
  |

2

mountain |  
 Kailash |  
 6638 |  
  |

2

mountain |  
 Mt. Kosciuszko |  
 2228 |  
 Highest point in Australia |  
  |

**Point locations with no elevation**

**Scale rank**

**Feature class** |  
 **Name** |  
 **Comment** |  
 **Alternative Name** |  
  
|  
  |

3

island |  
 Wright I. |  
  |

3

island |  
 Grant I. |  
  |

3

island |  
 Newman I. |  
  |

3

island |  
 Dean I. |  
  |

5

cape |  
 Cape Comorin |  
  |

5

cape |  
 Dondra Head |  
  |

5

cape |  
 Cabo Delgado |  
  |

5

cape |  
 Ponta da Barra |  
  |

5

cape |  
 Ponta São Sebastio |  
  |

5

cape |  
 Ras Cantin |  
  |

5

cape |  
 Ra’s Kasr |  
  |

5

cape |  
 Ra’s Banäs |  
  |

5

cape |  
 Ponta das Salinas |  
  |

5

cape |  
 Ponta das Palmeirinhas |  
  |

5

cape |  
 Cabo Bojador |  
  |

5

cape |  
 Ponta de Jericoacoara |  
  |

5

cape |  
 Cabo Orange |  
  |

5

cape |  
 Cabo de São Roque |  
  |

5

cape |  
 Ponta da Baleia |  
  |

5

cape |  
 Cabo de São Tomé |  
  |

5

cape |  
 Cabo Frio |  
  |

5

cape |  
 Cabo de Santa Marta Grande |  
  |

5

cape |  
 Punta del Este |  
  |

5

cape |  
 Cabo San Antonio |  
  |

5

cape |  
 Cabo Corrientes |  
  |

5

cape |  
 Punta Rasa |  
  |

5

cape |  
 Cabo San Diego |  
  |

5

cape |  
 Cabo Dos Bahías |  
  |

5

cape |  
 Cabo Tres Puntas |  
  |

5

cape |  
 Cape Yelizavety |  
  |

5

cape |  
 Pt. Yuzhnyy |  
  |

5

cape |  
 Cape Reinga |  
  |

5

cape |  
 South West Cape |  
  |

5

cape |  
 Cape Howe |  
  |

5

cape |  
 Cape Otway |  
  |

5

cape |  
 Cape Jaffa |  
  |

5

cape |  
 Cape Carnot |  
  |

5

cape |  
 Cape Byron |  
  |

5

cape |  
 Cape Manifold |  
  |

5

cape |  
 Cape York |  
  |

5

cape |  
 Cape Melville |  
  |

5

cape |  
 Cape Arnhem |  
  |

5

cape |  
 West Cape Howe |  
  |

5

cape |  
 Cape Leeuwin |  
  |

5

cape |  
 Cape Pasley |  
  |

5

cape |  
 Cape Londonderry |  
  |

5

cape |  
 Steep Point |  
  |

5

cape |  
 North West Cape |  
  |

5

cape |  
 Cape Cretin |  
  |

5

cape |  
 Cape Sata |  
  |

5

cape |  
 Cape Aniva |  
  |

5

cape |  
 Cape Terpeniya |  
  |

5

cape |  
 Cape Lopatka |  
  |

5

cape |  
 Cape Ozernoy |  
  |

5

cape |  
 Cape Olyutorskiy |  
  |

5

cape |  
 Cape Navarin |  
  |

5

cape |  
 Cape Lopatka |  
  |

5

cape |  
 Nordkapp |  
  |

5

cape |  
 Cabo de São Vicentete |  
  |

5

cape |  
 Cabo Fisterra |  
  |

5

cape |  
 Cape Ince |  
  |

5

cape |  
 Ra’s Fartak |  
  |

5

cape |  
 Ras Sharbatat |  
  |

5

cape |  
 Ra’s al Had |  
  |

5

cape |  
 Cap Saint André |  
  |

5

cape |  
 Cap Est |  
  |

5

cape |  
 Cape St. Lucia |  
  |

5

cape |  
 Cape St. Francis |  
  |

5

cape |  
 Cabo Gracias a Dios |  
  |

5

cape |  
 Cape San Blas |  
  |

5

cape |  
 Cape Brewster |  
  |

5

cape |  
 Cape Morris Jesup |  
  |

5

cape |  
 Cape Sable |  
  |

5

cape |  
 Cape Bauld |  
  |

5

cape |  
 Cape Fear |  
  |

5

island |  
 Kanton |  
  |

5

island |  
 Tabuaeran |  
  |

5

island |  
 Malden |  
  |

5

island |  
 Rarotonga |  
  |

5

island |  
 I. Robinson Crusoe |  
  |

5

island |  
 Rangiroa |  
  |

5

island |  
 Hachijjima |  
  |

5

island |  
 Pagan |  
  |

5

island |  
 Minamitori-shima |  
  |

5

island |  
 Wake I. |  
  |

5

island |  
 Îles Chesterfield |  
  |

5

island |  
 Funafuti |  
  |

5

island |  
 Tabiteuea |  
  |

5

island |  
 Aranuka |  
  |

5

island |  
 Nauru |  
  |

5

island |  
 Ebon |  
  |

5

island |  
 Jaluit |  
  |

5

island |  
 Mili |  
  |

5

island |  
 Majuro |  
  |

5

island |  
 Ailinglapalap |  
  |

5

island |  
 Kwajalein |  
  |

5

island |  
 Rongelap |  
  |

5

island |  
 Bikini |  
  |

5

island |  
 Is. Martin Vaz |  
  |

5

island |  
 Rockall |  
  |

5

island |  
 Grmsey |  
  |

5

island |  
 Surtsey |  
  |

5

island |  
 I. Guadalupe |  
  |

5

island |  
 St. Croix |  
  |

5

island |  
 Grand Cayman |  
  |

5

island |  
 I. de Cozumel |  
  |

5

island |  
 Miquelon |  
  |

5

island |  
 Bermuda Islands |  
  |

5

island |  
 San Salvador |  
  |

5

island |  
 Grenada |  
  |

5

island |  
 Barbuda |  
  |

5

island |  
 Antigua |  
  |

5

island |  
 Guadeloupe |  
  |

5

island |  
 Dominica |  
  |

5

island |  
 Martinique |  
  |

5

island |  
 Saint Lucia |  
  |

5

island |  
 Saint Vincent |  
  |

5

island |  
 Barbados |  
  |

5

island |  
 Tobago |  
  |

5

island |  
 Margarita |  
  |

5

island |  
 Curaao |  
  |

5

island |  
 Aruba |  
  |

4

cape |  
 Punta Negra |  
  |

4

cape |  
 Punta Galera |  
  |

4

cape |  
 Punta Lavapié |  
  |

4

cape |  
 Lands End |  
  |

4

cape |  
 Cape York |  
  |

4

cape |  
 Nunap Isua |  
  |

4

cape |  
 Cabo Corrientes |  
  |

4

cape |  
 Pt. Eugenia |  
  |

4

cape |  
 Cap Lopez |  
  |

4

cape |  
 Cape Agulhas |  
  |

4

cape |  
 Cape Bobaomby |  
  |

4

cape |  
 Cape Vohimena |  
  |

4

cape |  
 Point Conception |  
  |

4

cape |  
 Cape Hatteras |  
  |

4

cape |  
 Cape Sable |  
  |

4

cape |  
 Cape Hope |  
  |

4

cape |  
 Point Barrow |  
  |

4

cape |  
 Cap Blanc |  
  |

4

plain |  
 Plain of Jars |  
  |

4

island |  
 Vavau |  
  |

4

island |  
 I. de Pascua |  
  |

3

cape |  
 Cabo de Hornos |  
  |

3

cape |  
 Cape Canaveral |  
  |

3

cape |  
 Cape Mendocino |  
  |

3

cape |  
 Cabo San Lucas |  
  |

3

cape |  
 Cape Churchill |  
  |

3

cape |  
 Cape Cod |  
  |

3

cape |  
 Cape May |  
  |

3

cape |  
 Cape of Good Hope |  
  |

3

cape |  
 Gees Gwardafuy |  
  |

3

cape |  
 Cape Palmas |  
  |

3

cape |  
 Cape Verde |  
  |

3

cape |  
 Cap Bon |  
  |

3

pole |  
 Oceanic pole of inaccessibility |  
  |

3

pole |  
 South Magnetic Pole 2005 (est) |  
  |

3

pole |  
 North Magnetic Pole 2005 (est) |  
  |

3

waterfall |  
 Victoria Falls |  
  |

2

waterfall |  
 Salto Angel |  
  |

2

waterfall |  
 Iguazu Falls |  
  |

2

waterfall |  
 Niagara Falls |  
  |

**Marine areas**

Scale rank

Feature class |  
 Name |  
 Alternative name |  
  
|  
  |

0

ocean |  
 ARCTIC OCEAN |  
  |

0

ocean |  
 SOUTHERN OCEAN |  
  |

0

ocean |  
 NORTH ATLANTIC OCEAN |  
  |

0

ocean |  
 NORTH PACIFIC OCEAN |  
  |

0

ocean |  
 NORTH PACIFIC OCEAN |  
  |

0

ocean |  
 SOUTH PACIFIC OCEAN |  
  |

0

ocean |  
 SOUTH PACIFIC OCEAN |  
  |

0

ocean |  
 INDIAN OCEAN |  
  |

0

ocean |  
 SOUTH ATLANTIC OCEAN |  
  |

1

sea |  
 Black Sea |  
  |

1

sea |  
 Philippine Sea |  
  |

1

sea |  
 Coral Sea |  
  |

1

sea |  
 Tasman Sea |  
  |

1

bay |  
 Bay of Bengal |  
  |

1

sea |  
 South China Sea |  
  |

1

sea |  
 Sea of Japan |  
 East Sea |  
  
|  
  |

1

sea |  
 Mediterranean Sea |  
  |

1

sea |  
 Arabian Sea |  
  |

1

sea |  
 Beaufort Sea |  
  |

1

sea |  
 Caribbean Sea |  
  |

1

gulf |  
 Gulf of Mexico |  
  |

1

sea |  
 Labrador Sea |  
  |

1

bay |  
 Hudson Bay |  
  |

1

sea |  
 Caspian Sea |  
  |

1

bay |  
 Baffin Bay |  
  |

1

gulf |  
 Gulf of Alaska |  
  |

1

sea |  
 Red Sea |  
  |

1

sea |  
 Sea of Okhotsk |  
  |

1

sea |  
 Ross Sea |  
  |

1

sea |  
 Weddell Sea |  
  |

1

gulf |  
 Persian Gulf |  
 Arabian Gulf |  
  
|  
  |

1

sea |  
 Ross Sea |  
  |

2

sea |  
 Celebes Sea |  
  |

2

sea |  
 Sulu Sea |  
  |

2

sea |  
 Bering Sea |  
  |

2

sea |  
 Norwegian Sea |  
  |

2

sea |  
 Greenland Sea |  
  |

2

sea |  
 Banda Sea |  
  |

2

strait |  
 Luzon Strait |  
  |

2

bay |  
 Bay of Biscay |  
  |

2

channel |  
 Mozambique Channel |  
  |

2

gulf |  
 Gulf of Guinea |  
  |

2

sea |  
 Scotia Sea |  
  |

2

sea |  
 Baltic Sea |  
  |

2

sea |  
 Barents Sea |  
  |

2

sea |  
 North Sea |  
  |

2

channel |  
 Bristol Channel |  
  |

2

sea |  
 Inner Seas |  
  |

2

sea |  
 Irish Sea |  
  |

2

sea |  
 Java Sea |  
  |

2

sea |  
 Andaman Sea |  
  |

2

sea |  
 Yellow Sea |  
  |

2

sea |  
 East China Sea |  
  |

2

sea |  
 Chukchi Sea |  
  |

2

bay |  
 Bah’a de Campeche |  
  |

2

sea |  
 Arafura Sea |  
  |

2

sea |  
 Timor Sea |  
  |

2

gulf |  
 Gulf of Thailand |  
  |

2

sea |  
 Laccadive Sea |  
  |

2

sea |  
 Bellingshausen Sea |  
  |

2

sea |  
 Amundsen Sea |  
  |

2

strait |  
 Davis Strait |  
  |

2

sea |  
 Kara Sea |  
  |

2

sea |  
 Laptev Sea |  
  |

2

channel |  
 Drake Passage |  
  |

2

sea |  
 Mediterranean Sea |  
  |

3

gulf |  
 Great Australian Bight |  
  |

3

gulf |  
 Gulf of Aden |  
  |

3

gulf |  
 Gulf of Oman |  
  |

3

sea |  
 Tyrrhenian Sea |  
  |

3

sea |  
 White Sea |  
  |

3

sea |  
 Sea of Azov |  
  |

3

bay |  
 James Bay |  
  |

3

gulf |  
 Gulf of Carpentaria |  
  |

3

gulf |  
 Golfo de California |  
  |

3

strait |  
 Hudson Strait |  
  |

3

gulf |  
 Gulf of Finland |  
  |

3

channel |  
 English Channel |  
  |

3

gulf |  
 Gulf of Honduras |  
  |

3

gulf |  
 Golfe du Lion |  
  |

3

gulf |  
 Gulf of Bothnia |  
  |

3

sea |  
 Adriatic Sea |  
  |

4

strait |  
 Straits of Florida |  
  |

4

bay |  
 Bay of Plenty |  
  |

4

channel |  
 The North Western Passages |  
  |

7

sound |  
 Queen Charlotte Sound |  
  |

4

sea |  
 Ionian Sea |  
  |

4

gulf |  
 Gulf of St. Lawrence |  
  |

4

sea |  
 Molucca Sea |  
  |

4

sea |  
 Bismarck Sea |  
  |

4

sea |  
 Solomon Sea |  
  |

4

gulf |  
 Gulf of Tonkin |  
  |

4

strait |  
 Bering Strait |  
  |

4

bay |  
 Bay of Fundy |  
  |

4

gulf |  
 Río de la Plata |  
  |

4

strait |  
 Strait of Malacca |  
  |

4

strait |  
 Strait of Singapore |  
  |

4

strait |  
 Makassar Strait |  
  |

4

sea |  
 Ceram Sea |  
  |

4

strait |  
 Korea Strait |  
  |

4

sea |  
 Inner Sea |  
  |

4

strait |  
 Taiwan Strait |  
  |

4

bay |  
 Cook Inlet |  
  |

4

bay |  
 Bristol Bay |  
  |

4

bay |  
 Melville Bay |  
  |

4

gulf |  
 Gulf of Maine |  
  |

4

bay |  
 Chesapeake Bay |  
  |

4

strait |  
 Strait of Gibraltar |  
  |

4

gulf |  
 Golfo San Jorge |  
  |

4

sea |  
 Balearic Sea |  
  |

4

sea |  
 Aegean Sea |  
  |

4

gulf |  
 Gulf of Mannar |  
  |

4

gulf |  
 Shelikhova Gulf |  
  |

4

sea |  
 Bo Hai |  
  |

4

gulf |  
 Gulf of Kutch |  
  |

4

gulf |  
 Golfo de Panamá |  
  |

4

gulf |  
 Amundsen Gulf |  
  |

4

sound |  
 Viscount Melville Sound |  
  |

4

bay |  
 Ungava Bay |  
  |

4

reef |  
 Great Barrier Reef |  
  |

5

channel |  
 Yucatan Channel |  
  |

5

sea |  
 Bering Sea |  
  |

5

sea |  
 East Siberian Sea |  
  |

5

sea |  
 Lincoln Sea |  
  |

5

strait |  
 Bass Strait |  
  |

5

bay |  
 Franklin Bay |  
  |

5

channel |  
 Dixon Entrance |  
  |

5

strait |  
 M’Clure Strait |  
  |

5

bay |  
 Boca Grande |  
  |

5

bay |  
 Baía de Marajó |  
  |

5

gulf |  
 Golfo San Mat’as |  
  |

5

gulf |  
 Golfo Corcovado |  
  |

5

gulf |  
 Wrigley Gulf |  
  |

5

bay |  
 Sulzberger Bay |  
  |

5

channel |  
 Ronne Entrance |  
  |

5

strait |  
 Bransfield Str. |  
  |

5

sound |  
 McMurdo Sound |  
  |

5

bay |  
 Baía de São Marcos |  
  |

5

sound |  
 Cumberland Sd. |  
  |

5

bay |  
 Frobisher Bay |  
  |

5

bay |  
 Disko Bay |  
  |

5

bay |  
 Hamilton Inlet |  
  |

5

fjord |  
 Storfjorden |  
  |

5

fjord |  
 Vestfjorden |  
  |

5

fjord |  
 Skagerrak |  
  |

5

strait |  
 Karskiye Vorota Strait |  
  |

5

strait |  
 Vil’kitskogo Strait |  
  |

5

fjord |  
 Sognefjorden |  
  |

5

fjord |  
 Trondheimsfjorden |  
  |

5

sea |  
 Kattegat |  
  |

5

gulf |  
 Gulf of Khambhät |  
  |

5

strait |  
 Palk Strait |  
  |

5

gulf |  
 Kronotskiy Gulf |  
  |

5

bay |  
 Uda Bay |  
  |

5

bay |  
 Uchiura Bay |  
  |

5

strait |  
 Tsugaru Strait |  
  |

5

strait |  
 Tatar Strait |  
  |

5

bay |  
 Shark Bay |  
  |

5

gulf |  
 Gulf of Gabès |  
  |

5

bay |  
 Mackenzie Bay |  
  |

5

sound |  
 Norton Sound |  
  |

5

gulf |  
 Gulf of Anadyr’ |  
  |

5

sound |  
 Kotzebue Sound |  
  |

5

gulf |  
 Gulf of Boothia |  
  |

5

bay |  
 Kane Basin |  
  |

5

bay |  
 Foxe Basin |  
  |

5

gulf |  
 Golfo de Tehuantepec |  
  |

5

gulf |  
 Golfo de Guayaquil |  
  |

5

gulf |  
 Gulf of Masira |  
  |

5

gulf |  
 Gulf of Yana |  
  |

5

strait |  
 Dmitriy Laptev Strait |  
  |

5

strait |  
 La Perouse Strait |  
  |

5

bay |  
 East Korea Bay |  
  |

5

strait |  
 Qiongzhou Strait |  
 Hainan Strait |  
  
|  
  |

5

strait |  
 Cook Strait |  
  |

5

strait |  
 Torres Strait |  
  |

5

bay |  
 Geographe Bay |  
  |

5

gulf |  
 Gulf of Papua |  
  |

5

gulf |  
 Gulf of Olen‘k |  
  |

5

gulf |  
 Gulf of Riga |  
  |

5

lagoon |  
 IJsselmeer |  
  |

5

gulf |  
 Gulf of Sidra |  
  |

5

sea |  
 Sea of Crete |  
  |

5

sea |  
 Ligurian Sea |  
  |

5

sea |  
 Alboran Sea |  
  |

5

bay |  
 Bight of Benin |  
  |

5

bay |  
 Bight of Biafra |  
  |

5

strait |  
 Strait of Belle Isle |  
  |

5

strait |  
 Strait of Juan de Fuca |  
  |

5

sound |  
 Prince William Sound |  
  |

5

strait |  
 Estrecho de Magellanes |  
  |

5

bay |  
 Bahía Blanca |  
  |

5

gulf |  
 Golfo de Penas |  
  |

5

bay |  
 Bahía Grande |  
  |

5

bay |  
 Marguerite Bay |  
  |

5

bay |  
 Prydz Bay |  
  |

5

bay |  
 Vincennes Bay |  
  |

5

bay |  
 Porpoise Bay |  
  |

5

sea |  
 Davis Sea |  
  |

5

bay |  
 Lützow-Holm Bay |  
  |

5

bay |  
 Helodranon’ Antongila |  
  |

5

bay |  
 Hangzhou Bay |  
  |

5

gulf |  
 Karaginskiy Gulf |  
  |

5

gulf |  
 Gulf of Kamchatka |  
  |

5

strait |  
 Bosporus |  
  |

5

strait |  
 Bab el Mandeb |  
  |

5

gulf |  
 Gulf St. Vincent |  
  |

5

gulf |  
 Golfo de Urabá |  
  |

5

bay |  
 Baia de Maputo |  
  |

5

bay |  
 St. Helena Bay |  
  |

5

fjord |  
 Boknafjorden |  
  |

5

gulf |  
 Joseph Bonaparte Gulf |  
  |

5

gulf |  
 Gulf of Martaban |  
  |

5

gulf |  
 Gulf of Sakhalin |  
  |

5

fjord |  
 Kangertittivaq |  
 Scoresbysund |  
  
|  
  |

6

channel |  
 St. Lawrence River |  
  |

6

bay |  
 Chaun Bay |  
  |

6

gulf |  
 Khatanga Gulf |  
  |

6

bay |  
 Garabogaz Bay |  
  |

6

gulf |  
 Gulf of Suez |  
  |

6

gulf |  
 Gulf of Ob |  
  |

6

gulf |  
 Yenisey Gulf |  
  |

6

channel |  
 Dardanelles |  
  |

6

sea |  
 Sea of Marmara |  
  |

6

gulf |  
 Gulf of Aqaba |  
  |

6

lagoon |  
 Lago de Maracaibo |  
  |

6

strait |  
 Denmark Strait |  
  |

7

bay |  
 Massachusetts Bay |  
  |

7

bay |  
 Delaware Bay |  
  |

7

sound |  
 Long Island Sound |  
  |

7

sound |  
 Albemarle Sound |  
  |

7

sound |  
 Pamlico Sound |  
  |

7

sound |  
 Puget Sound |  
  |

7

strait |  
 Strait of Georgia |  
  |

This entry was posted
on Monday, September 21st, 2009 at 8:30 am and is filed under 50m-physical-vectors.
You can follow any responses to this entry through the RSS 2.0 feed.
Both comments and pings are currently closed.


Comments are closed.

- Subscribe: Entries | Comments
- ## Search

  Search for:
- ## Links

  - NACIS
- ## Tags

  10m
  50m
  90
  180
  admin-0
  bjorn
  bounding box
  browser
  change log
  corrections
  countries
  Downloads
  error
  extent
  ext js
  forums
  geoext
  hans
  imagery
  import
  mapnik
  maptiler
  map tiles
  marine boundary
  national parks
  new data
  nsd
  openlayers
  physical labels
  pngng
  populated places
  raster
  terrestrial hypsography
  tfw
  thematic mapping
  themese
  tif
  tilecache
  tiles
  time zones
  towns
  transportation
  update
  visitors
  world file
- ## Recent Comments

  - ivermectin 3mg tablets on Download URLs – double slash
  - stromectol 6 mg dosage on Download URLs – double slash
  - Avenza Systems | Map and Cartography Tools on 1:50m Manual Shaded Relief
  - ivermectin 6mg tablets on Download URLs – double slash
  - stromectol cvs on Download URLs – double slash
- ## Recent Forum Topics

  - Natural Earth in Wagner VII
    by Hugo Ahlenius
  - Downloads are 404ing
    by Nathaniel
  - Disputed Territories: "type" field
    by alykat
  - ISO code confusion
    by nth
  - Bad ADM1NAME, encoding in version 3.0.0 and missing diacritics in NAME
    by pfunes
  - U.S. County Shape File
    by gzingsheim
  - Projection / Proportion / Compatibility?
    by Liquidized
  - Download URLs – double slash
    by vastur
  - map soft – writer: me
    by krzysztof
  - Unicode encoding issue – ne\_10m\_lakes.dbf
    by filter.1

---

Supported by:

© 2009 - 2021. Natural Earth. All rights reserved.


Powered by WordPress

Staff Login »
