# Supplementary material for: Potential patterns of fall armyworm seasonal migration in West Africa modeled with atmospheric trajectory analyses
Source: Pest Manag Sci. 2026 Mar 22;82(7):6509–17. doi: 10.1002/ps.70732 (PMC13240689; doi:10.1002/ps.70732)
Supplement: Supplementary file 2 — Data S2. Supporting Information. [file PS-82-6509-s002.zip › 3_sup 2.zip!/Supplementary Material2/raw data/Supplementary Material 2 Description.doc]

**List of correspondences between file paths in the scripts and uploaded file names**

**Note:** Due to the large volume of data, we provide the raw trajectory data at 5-hour intervals over a 3-day period (highlight in blue) for reference.The scripts include all code used to generate the results and supplementary figures. Each script name corresponds to the results it produces. The scripts should be run in the following order:

Result1.R (produces Fig. 2)

FigureS1_2_and_Result2.1.R (produces Figs. S1, S2 and Fig. 3)

Result2.2.R (produces Fig. 4)

Result3.R (produces Fig. 5)

Result4.R (produces Fig. 6)

| **G:/trajactory simulation/FAW suitable habitats in Africa shp/Mean contour.shp** | **Mean contour.shp** |
| --- | --- |
| **G:/niches shift/data/boundary data/global boundary/ne_10m_admin_0_countries_chn.shp** | **ne_10m_admin_0_countries_chn.shp** |
| **G:/trajactory simulation/desert shp/ne_50m_geography_regions_polys.shp** | **ne_50m_geography_regions_polys.shp** |
| **G:/trajactory simulation/take off/take off points all.csv** | **take off points all.csv** |
| **G:/trajactory simulation/2021 result/medium1.txt** | **2021 result/medium1.txt** |
| **G:/trajactory simulation/2021 result/medium2.txt** | **2021 result/medium2.txt** |
| **G:/trajactory simulation/2022 result/medium1.txt** | **2022 result/medium1.txt** |
| **G:/trajactory simulation/2022 result/medium2.txt** | **2022 result/medium2.txt** |
| **G:/trajactory simulation/2023 result/medium1.txt** | **2023 result/medium1.txt** |
| **G:/trajactory simulation/2023 result/medium2.txt** | **2023 result/medium2.txt** |
| **G:/trajactory simulation/three year landing points result/summary table.csv** | **summary table.csv** |
| **G:/trajactory simulation/wind/data.grib** | **wind data.grib** |
